# Unraveling Targetable Systemic and Cell-Type-Specific Molecular Phenotypes of Alzheimer’s and Parkinson’s Brains With Digital Cytometry

**DOI:** 10.3389/fnins.2020.607215

**Published:** 2020-12-09

**Authors:** Marie C. Bordone, Nuno L. Barbosa-Morais

**Affiliations:** Instituto de Medicina Molecular João Lobo Antunes, Faculdade de Medicina, Universidade de Lisboa, Lisbon, Portugal

**Keywords:** Alzheimer’s disease, Parkinson’s disease, neurodegeneration, cellular deconvolution, digital cytometry, single-cell RNA-seq, chemo-transcriptomics

## Abstract

Alzheimer’s disease (AD) and Parkinson’s disease (PD) are the two most common neurodegenerative disorders worldwide, with age being their major risk factor. The increasing worldwide life expectancy, together with the scarcity of available treatment choices, makes it thus pressing to find the molecular basis of AD and PD so that the causing mechanisms can be targeted. To study these mechanisms, gene expression profiles have been compared between diseased and control brain tissues. However, this approach is limited by mRNA expression profiles derived for brain tissues highly reflecting their degeneration in cellular composition but not necessarily disease-related molecular states. We therefore propose to account for cell type composition when comparing transcriptomes of healthy and diseased brain samples, so that the loss of neurons can be decoupled from pathology-associated molecular effects. This approach allowed us to identify genes and pathways putatively altered systemically and in a cell-type-dependent manner in AD and PD brains. Moreover, using chemical perturbagen data, we computationally identified candidate small molecules for specifically targeting the profiled AD/PD-associated molecular alterations. Our approach therefore not only brings new insights into the disease-specific and common molecular etiologies of AD and PD but also, in these realms, foster the discovery of more specific targets for functional and therapeutic exploration.

## Introduction

Alzheimer’s disease (AD) and Parkinson’s disease (PD) are the two most common neurodegenerative disorders worldwide. Although the etiology, affected brain region and clinical features are particular to each of these diseases, they nevertheless share common mechanisms such as mitochondria dysfunction, neuronal loss and tau protein accumulation (Zarow et al., 2003; Xie et al., 2014). The major risk factor for those disorders is aging (Niccoli and Partridge, 2012), the age of onset of both AD or PD being around 65 years old (Nussbaum and Ellis, 2003). Together, they account for 50 million cases worldwide (Feigin et al., 2017), a number expected to increase due to the fact that the world population is living longer than ever (Goedert, 2015; World Health Organization, 2020). Most of AD and PD cases are sporadic and, despite all the research during the last centuries to better understand their molecular nature, current treatments are still symptomatic (Yiannopoulou and Papageorgiou, 2013; Schulz et al., 2016). Therefore, the development of effective therapies requires a better comprehension of the diseases’ etiology and underlying mechanisms as well as finding disease-specific targets for drug discovery.

A common strategy to identify biological pathways and cellular processes altered in neurodegenerative disorders is to compare gene expression profiles between age-matched diseased and non-diseased *post-mortem* brain tissues (Wang et al., 2009; Srinivasan et al., 2016) or between diseased and non-diseased leukocytes (Soreq et al., 2013, 2014). However, the expression profiles derived from whole brain tissue mRNA highly reflect alterations in cellular composition, namely the well-known AD- or PD-associated loss of neurons, but not necessarily the disease-related molecular changes in brain cells (Srinivasan et al., 2016). The advent of single-cell transcriptomes has made it possible to tackle this limitation, enabling the determination of reference gene expression profiles for each major brain cell type (namely neurons, astrocytes, microglia and oligodendrocytes) (Darmanis et al., 2015; Lake et al., 2016, 2018; Mathys et al., 2019a; Schirmer et al., 2019; Velmeshev et al., 2019) that can then be used to computationally estimate the cell type-specific content of bulk brain sample’s in healthy and diseased conditions (Kuhn et al., 2012, 2015), decoupling the neuronal loss effect from the intrinsic systemic or cell type-specific disease effects (Capurro et al., 2015; Skene and Grant, 2016).

This approach has already been applied in determining the effects of age and psychiatric disorders on the cellular composition of human brain (Hagenauer et al., 2017), or the contribution of each cell type in shaping the pathological autism transcriptome (Yu and He, 2017). The same principle was applied in AD by modeling the expression of its risk genes as a function of estimated cellular composition of brain samples (Kelley et al., 2018). For instance, *APP*, *PSEN1*, *APOE*, and *TREM2* had their expression levels associated with the relative abundance of, respectively, neurons, oligodendrocytes, astrocytes and microglia (Kelley et al., 2018). Additionally, two recent studies profiled single nuclei of major brain cell types in AD and non-AD *post-mortem* brain samples, unveiling cell type-specific transcriptional changes (Mathys et al., 2019a; Zhou et al., 2020). All these studies highlight the importance of charactering disease-associated cell type-specific phenotypes that can not only unveil the cellular and molecular bases of pathological mechanisms but also be therapeutically targeted.

However, some of these studies still lack independent validation and have not fully dissected the nature of transcriptomic alterations in AD brains. Moreover, to our knowledge, similar approaches have not yet been applied to PD, despite increasing evidence regarding the importance of modeling cellular composition in neurodegenerative disorders. We therefore used scRNA-seq data to derive gene expression signatures for the major human brain cell types (Darmanis et al., 2015) and estimate the cellular composition of idiopathic AD (Allen et al., 2016) and PD (Dumitriu et al., 2016) *post-mortem* brain samples from their bulk transcriptomes, investigating whether neuronal loss could be confounding or masking the intrinsic disease effects on gene expression, and validating the results in independent datasets. Additionally, since AD and PD might share the same mechanisms of disease progression (Nussbaum and Ellis, 2003), we also investigated the similarities between the transcriptomic alterations induced by AD and PD in human brain tissues.

This approach allowed the novel identification of genes and pathways whose activity in the brain is intrinsically altered by AD and PD in systemic and cell type-specific ways. Additionally, we pinpoint the genes that are commonly altered by these major neurodegenerative disorders as well as those specifically perturbed in each illness. Thus, we unveil a set of novel candidates that can potentially be targeted in AD and PD therapeutics. Moreover, we herein demonstrate the potential of modeling cellular composition in transcriptomics analyses in the discovery of therapeutic targets for other neurodegenerative diseases.

## Materials and Methods

### Data Availability

We obtained, through NCBI Gene Expression Omnibus (GEO), two single-cell RNA-seq datasets that we employed to derive gene expression signatures for the major brain cell types (i.e., astrocytes, microglia, neurons and oligodendrocytes), one from human temporal lobe [GSE67835 (Darmanis et al., 2015) – only cells from adult samples were used] and the other from mouse cortex [SRP135960 (Zeisel et al., 2015)]. We used a third single-cell RNA-seq dataset [GEO GSE73721 (Zhang et al., 2016)] to independently validate those signatures, considering only cells from the human cortex (12 astrocytes, 1 neuron, 4 oligodendrocytes, and 2 endothelial cells), for consistency.

The AD analysis was based on the temporal cortex RNA-seq dataset from the AMP-AD Knowledge Portal with accession syn3163039 (Allen et al., 2016). We used the table available therein, containing the pre-processed raw read counts for each gene in each sample, for the downstream analyses. We selected only the samples diagnosed as AD and non-AD with RNA integrity number (RIN) ≥8 (Ferrer et al., 2008). We also discarded a non-AD sample with a very low (<0.40) estimated proportion of neurons (Supplementary Figure 1A and Supplementary Table 1). In total, we used 71 AD and 32 non-AD samples.

We used AD dataset GEO GSE104704 (Nativio et al., 2018) for independent validation, less stringently requiring RIN ≥6 to keep enough samples for analysis. Three non-AD samples with abnormally low (<0.40) estimated proportion of neurons were discarded (Supplementary Figure 1B and Supplementary Table 1), leaving a total of 9 AD and 14 non-AD samples.

We fetched the PD RNA-seq dataset from GEO GSE68719 (Dumitriu et al., 2016) and kept samples with RIN ≥7 and from donors older than 60 years, for a better age match between control and diseased samples and given the reported onset of idiopathic PD at around 65 years of age (Nussbaum and Ellis, 2003). When performing principal component analysis (PCA) of the normalized gene expression data (see sections “Statistical Tests” and “Data Processing” below), we identified two samples (SRR2015728 and SRR2015748) with an outlying behavior (Supplementary Figures 2A,B). When clustering samples based on the correlation between their normalized gene expression profiles (see section “Statistical Tests” below), SRR2015728 and SRR2015748 are again shown to be outliers (Supplementary Figure 2C). Moreover, non-PD samples SRR2015714 and SRR2015728 are also those showing an abnormally low (<0.40) estimated neuronal proportion (Supplementary Figure 1C and Supplementary Table 1). As such, we conservatively discarded those three samples from the dataset, leaving 15 PD and 26 non-PD samples.

For independent validation, we used PD gene expression microarray dataset GEO GSE20168 (Zhang et al., 2005). Since the PD RNA-seq dataset only comprised males, we selected the 10 non-diseased and 8 PD male samples from the microarray dataset. Although RINs were not provided for this dataset, we were able to detect possible RNA degradation by using function *AffyRNAdeg* from the *xps* R package (Stratowa, 2020). We found some evidence for the expected neuronal loss in PD brains but differences in the distributions of estimated neuronal proportions between PD and non-PD samples are not statistically significant (Supplementary Figure 1D). Although one non-PD sample had a low (<0.40) estimated neuronal proportion, we decided to keep it due to the small number of samples in the dataset (Supplementary Table S1).

All datasets used are summarized in Table 1.

**TABLE 1:** Summary of transcriptomic datasets analyzed.

	Accession	Species	Type	Condition	Technology	Pre-processed data
**Darmanis** (Darmanis et al., 2015)	GEO GSE67835	Human	Single cell	Normal	RNA-seq	No
**Mouse** (Zeisel et al., 2018)	http://mousebrain.org/downloads.html	Mouse	Single cell	Normal	RNA-seq	Yes
**Zhang** (Zhang et al., 2016)	GEO GSE73721	Human	Single cell	Normal	RNA-seq	No
**MayoClinic** (Allen et al., 2016)	https://www.synapse.org/#!Synapse: syn3163039	Human	Bulk	AD + Normal	RNA-seq	Yes
**Nativio** (Nativio et al., 2018)	GEO GSE104704	Human	Bulk	AD + Normal	RNA-seq	No
**Dumitriu** (Dumitriu et al., 2016)	GEO GSE68719	Human	Bulk	PD + Normal	RNA-seq	No
**Zhang bulk** (Zhang et al., 2005)	GEO GSE20168	Human	Bulk	PD + Normal	Microarray (Affymetrix Human Genome U133A)	No

### Statistical Tests

We performed all statistical analyses in R (programming language for statistics and graphics) (R Development Core Team, 2018), extensively using packages from Bioconductor (repository of R tools for the analysis of high-throughput biological data) (Gentleman et al., 2004). We used *t*-tests (Kalpić et al., 2011) to compare differences in expression of specific marker genes, as well as differences in age distributions between diseased and non-diseased groups. To compare differences in proportions of neural cell types between diseased and non-diseased brains, we used Wilcoxon-signed-rank tests (Rey and Neuhäuser, 2011), and to compare the neuronal proportion densities between diseased and non-diseased brains, we used the Kolmogorov-Smirnov test (Lopes, 2011). For correlation analysis, we used Pearson’s correlation, unless stated otherwise. We chose Euclidean distance for clustering samples based on gene expression correlation, having used the *ComplexHeatmap* package (Gu et al., 2016) for the purpose and to generate the associated heatmap in Supplementary Figure 2C.

Principal component analysis (PCA), enabling the identification of the linear combinations of variables that contribute the most to data variance (Ringnér, 2008), was implemented through the singular value decomposition (SVD) algorithm provided by the *PCA* function from R package *FactoMineR* (Lê et al., 2008).

Where applicable and not indicated otherwise, *p*-values were corrected for multiple testing using Benjamin-Hochberg’s False Discovery Rate (FDR).

### Data Processing

For all the RNA-Seq datasets with no pre-processed data available (Table 1), we aligned the reads against the human transcriptome [hg38 Gencode annotation (Frankish et al., 2019)] with Kallisto (Bray et al., 2016) using the default parameters.

For both single-cell datasets, we performed state-of-the-art procedures for quality assessment (Lun et al., 2016b), such as checking for library size discrepancies between cells, the number of expressed genes per cell and the proportion of reads aligning to mitochondrial genes (Lun et al., 2016b; Supplementary Figures 3A,C). We removed low-quality cells that presented a median absolute deviation (MAD) <−3 for the library size, MAD < −3 for the number of expressed genes or a MAD > 3 for the proportion of mitochondrial reads. Additionally, we kept for downstream analysis only genes whose log_10_[average read counts per million (CPM)] > 0 (Supplementary Figures 3B,D) and whose variance in expression was significantly associated with the biological component (i.e., the cell type) as assessed through the usage of the *decomposeVar* function from the *scran* R package (Lun et al., 2016b). Briefly, the variance in expression for each gene was decomposed into their biological and technical components. The technical component is estimated by fitting the mean-dependent trend of the variance. The biological component of the variance is then calculated by subtracting the technical component from the overall variance (Lun et al., 2016b). This last step avoids prioritizing genes whose expression is highly variable due to technical factors such as sampling noise during RNA capture and library preparation (Lun et al., 2016b).

Furthermore, the t-Distributed Stochastic Neighbor Embedding (tSNE) (van der Maaten and Hinton, 2008) plot of human single-cell (Darmanis) gene expression shows a few cells not clustered together with those of their respective annotated type (Supplementary Figure 4A). Moreover, all of them appear to have been misclassified also based on single-cell trajectories [i.e., cells’ ordering according to their inferred biological state (Qiu et al., 2017b)] obtained with the *monocle* package (Trapnell et al., 2014; Qiu et al., 2017a, b; Supplementary Figure 4B) or the nearest shrunken centroid classification, implemented in R package *pamr* (Tibshirani et al., 2002; Supplementary Figures 4C,D). Therefore, they were discarded from our analysis (Supplementary Figure 4E). No potentially misclassified cells were detected in the mouse dataset (Supplementary Figure 4F).

After the filtering steps mentioned above, summed expression values across pools of cells were deconvolved in cell-based factors for normalization of the Darmanis and the mouse single-cell gene expression datasets (Lun et al., 2016a). All bulk RNA-seq datasets were quantile-normalized using the *voom* function, from the *limma* R package (Ritchie et al., 2015). The *rma* function from the *affy* R package (Gautier et al., 2004) was used to normalize and summarize the PD microarray dataset.

Moreover, we used the *ComBat* function from the *sva* package to correct for batch effects. This function requires possible technical effects to be encoded as categorical variables (Leek et al., 2012). Thus, for the AD MayoClinic dataset, RIN was defined as high if >8.5 and low if ≤8.5, in the AD Nativio dataset high if >7.3 and low if ≤7.3, and in the PD Dumitriu dataset it was defined as high if >7.8 and low if ≤7.8. For the PD Zhang dataset, the RNA degradation slope, derived from the average intensities per relative 5′–3′ position of probes in their target transcripts across probe sets (Draghici, 2012), was used and defined as low if ≤5 and high if >5.

We quantified gene expression from RNA-seq data in counts per million (CPM) and kept only genes with an average CPM higher of 10*/L*, where *L* is the minimum library size in million reads (Chen et al., 2016), in at least *N* samples, where *N* is the smallest sample size in our analyses (Supplementary Figure 5). For the microarray dataset, gene expression was quantified by normalized intensities.

### Derivation of Gene Expression Signatures for the Major Brain Cell Types

We employed CIBERSORTx (Newman et al., 2019) to infer both human and mouse gene expression signatures for each of the major brain cell types (Supplementary Tables 2, 3) and subsequently used them to estimate the cellular composition of brain samples from their bulk transcriptomes.

We followed three different approaches to assess the accuracy of human and mouse CIBERSORTx-derived signatures in correctly identifying the major cell types in human brain samples:

(1) We split the Darmanis human dataset such that 80% of cells were used to infer cell type-specific gene expression signatures with CIBERSORTx. We used the remaining 20% of cells, with the same proportion of each cell type, to create 300 artificial mixture samples with a diverse range of known (i.e., pre-defined) cell-type proportions (Supplementary Figure 6A) by generating chimeric libraries of 35 million reads. In brief, all the reads from all cells of each cell type were pooled together. For each artificial sample, reads were randomly sampled from cell-type-specific pools according to its defined cell type proportion as in Supplementary Table 4. We treated the artificial mixture samples as bulk RNA-seq samples.
  CIBERSORTx estimated the cell type proportions of the artificial mixtures, using the human (Darmanis) cell-type-specific gene expression signatures. Those estimates are generally concordant with the expected proportions, except for the systematic underestimation of microglia’s relative abundance (Supplementary Figure 6B). We repeated the deconvolution analysis in the same artificial mixtures but using the mouse cell-type signatures and got a similar, albeit noisier, concordance (Supplementary Figure 6C).
(2) We ran CIBERSORTx using the same human and mouse cell-type signatures, to classify samples from an independent human brain single-cell RNA-seq dataset (Zhang). Most cells were correctly classified with the human signature (Supplementary Figure 7A). With the mouse signature, most cells are classified as a mixture of cell types but with a dominant proportion of that expected (Supplementary Figure 7C).
(3) We generated artificial mixtures from the Zhang dataset as in (1). Those artificial mixtures were then deconvoluted with CIBERSORTx relying again on the derived human and mouse signatures. Both signatures yield significant concordances (all with *p* < 2.2e-16) between the expected and observed proportions, with the human signature being again, as expected, more accurate (Supplementary Figures 7B,D).

### Estimation of Cellular Composition of Bulk AD, PD, and Non-diseased Brain Samples

We used CIBERSORTx deconvolution (Newman et al., 2019), relying on human and mouse gene expression signatures for the major brain cell types derived as described above, to estimate the cellular composition of all AD, PD, and non-diseased brain samples from their bulk transcriptomes. Moreover, as CIBERSORTx options, we enabled batch normalization, disabled quantile normalization and used 100 permutations for significance analysis. Following CIBERSORTx’s user guidelines, the B-mode batch normalization was chosen to perform deconvolution when using the human signature, since the single cell data used to derive it were generated with SMART-seq2 (Picelli et al., 2013), and the S-mode batch normalization when using the mouse signature, since it is tailored for signatures derived from data generated with the 10x Genomics Chromium platform, as was the case (Newman et al., 2019).

### Differential Gene Expression

We performed differential gene expression using the *limma* (Ritchie et al., 2015) and *edgeR* (Dai et al., 2014) packages.

For each coefficient in the linear model, the magnitude of differences in gene expression was measured in log_2_ fold-change and their significance was given by the FDR-adjusted *p*-value of the moderated *t*-statistic (an ordinary t-statistic with its standard errors moderated across genes), along with the empirical Bayes statistic (B statistic - log-odds ratio of a gene being differentially expressed) (Smyth, 2004). Moreover, we also used the moderated t-statistic to assess the differential gene expression coherence between different datasets.

We linearly modeled gene expression in the AD datasets according to the following:

$$G\,E_{x}=\beta_{0}+\beta_{D\,i\,s\,e\,a\,s\,e}•D\,i\,s\,e\,a\,s\,e+\beta_{R\,I\,N}•R\,I\,N+\beta_{N\,e\,u\,r\,o\,n\,a\,l\,\,p\,r\,o\,p\,o\,r\,t\,i\,o\,n}$$

$$•N\,e\,u\,r\,o\,n\,a\,l\,\,p\,r\,o\,p\,o\,r\,t\,i\,o\,n+\beta_{A\,g\,e}•A\,g\,e+\beta_{s\,e\,x}•S\,e\,x+\beta_{I\,n\,t\,e\,r\,a\,c\,t\,i\,o\,n}•I\,n\,t\,e\,r\,a\,c\,t\,i\,o\,n+\varepsilon$$

Here *GE*_*x*_ is the expression of gene x; *Disease* is the sample’s centered disease status; *RIN* is the categorized sample’s RNA Integrity Number (1 for high and 0 for low); *Neuronal proportion* is given by the sample’s estimated proportion of neurons centered; *Age* is the age of the sample’s donor in years; *Sex* is the biological sex of the sample’s donor (1 for male and 0 for female); *Interaction* is the interaction between *Disease* and the *Neuronal proportion* effects, given by the product of the two and interpretable as the differential effect of the loss of neurons between AD and non-diseased samples or, equivalently, the part of AD effect that is dependent of the sample’s neuronal contents; βs are the unknown coefficients, to be estimated from fitting that linear model to the gene expression data, for each of the aforementioned variables hypothesized to impact gene expression; ε states the error of the model, that is the remaining variance not explained by the model. *Disease* and *Neuronal proportion* were centered to diminish the correlation between their associated estimated coefficients, thereby using a model more consistent with the purpose of estimating independent effects (Afshartous and Preston, 2011). We thus shifted the “prediction center” (i.e., the virtual reference) to the average sample (Afshartous and Preston, 2011) by turning the variables’ means to 0 through the usage of the *scale* function from the built-in R package *base* (R Development Core Team, 2018), with the *scale* argument turned to “false.”

Likewise, we modeled gene expression in the PD Dumitriu dataset as following:

$$G\,E_{x}=\beta_{0}+\beta_{D\,i\,s\,e\,a\,s\,e}•D\,i\,s\,e\,a\,s\,e+\beta_{R\,I\,N}•R\,I\,N+\beta_{N\,e\,u\,r\,o\,n\,a\,l\,\,p\,r\,o\,p\,o\,r\,t\,i\,o\,n}$$

$$•N\,e\,u\,r\,o\,n\,a\,l\,\,p\,r\,o\,p\,o\,r\,t\,i\,o\,n+\beta_{A\,g\,e}•A\,g\,e+\beta_{U\,n\,k\,n\,o\,w\,n\,\,b\,a\,t\,c\,h}•U\,n\,k\,n\,o\,w\,n\,\,b\,a\,t\,c\,h+\varepsilon$$

*Unknown batch* corresponds to a batch effect of unknown source detected by PCA (Supplementary Figure S8) that was thereby adjusted for (Supplementary Figure 12C).

For validation with the independent PD microarray dataset (Zhang), we used the following linear model:

$$G\,E_{x}=\beta_{0}+\beta_{D\,i\,s\,e\,a\,s\,e}•D\,i\,s\,e\,a\,s\,e+\beta_{N\,e\,u\,r\,o\,n\,a\,l\,\,p\,r\,o\,p\,o\,r\,t\,i\,o\,n}$$

$$•N\,e\,u\,r\,o\,n\,a\,l\,\,p\,r\,o\,p\,o\,r\,t\,i\,o\,n+\beta_{A\,g\,e}•A\,g\,e+\beta_{R\,N\,A\,\,\mathrm{de}\,g\,r\,a\,d\,a\,t\,i\,o\,n}•R\,N\,A\,\deg\,r\,a\,d\,a\,t\,i\,o\,n+\varepsilon$$

*RNA degradation* is given by its slope grouping for each sample (1 for high and 0 for low).

We considered a gene differentially expressed if FDR < 0.05, except for the Zhang PD microarray dataset, where we considered FDR < 0.11. This arbitrary cut-off was used to “rescue” a reasonable number genes for further analyses, given the small sample size of the Zhang dataset and the consequent lower statistical power of the associated differential expression analysis. This arbitrary looseness in specificity is dealt with by subsequent filtering (v. section on permutation analyses below).

### Identification of Genes Reportedly Associated With AD and PD

Genes already reported to play a role in AD and PD were gathered from the DisGeNET database (Piñero et al., 2019). Only genes with a human gene-disease association (GDA) score >0.1 and an evidence index ≥0.9 (180 genes for AD and 112 genes for PD) were considered as such in our analyses.

### Permutation Analyses

We performed permutation tests to identify genes with consistent differential expression ranking between datasets. For each gene, we multiplied its *t*-statistic values for the intrinsic disease effect (*Disease* in the linear models) in each of the two datasets (MayoClinic and Nativio for AD; Dumitriu and Zhang for PD) and compared that product with the distribution of those resulting from 5000 random permutations of the disease status labeling of samples. The proportion of random products more extreme than the empirical one was taken as its False Discovery Rate (FDR).

To assess the similarity between the intrinsic AD and PD *Disease* effects on gene expression, we compared the aforementioned FDRs. For each disease, when *t*-statistics for both datasets were positive, we used −log_10_(FDR), when both negative, we used log10(FDR), and when contradictory (i.e., showing different signs) we set this value to 0. When FDRs were originally zero, we equaled them to 1e-5 (half of the FDR resolution) to avoid infinite values when computing their logarithms. Then, for each gene, we multiplied those scores of AD and PD and compared this product with the distribution yielded by 1 000 000 permutations of randomly shuffled product scores. The proportion of random products more extreme than the empirical one was taken as its FDR (Figure 7A).

### Gene Set Enrichment Analysis

We identified KEGG (Kanehisa et al., 2016) pathways dysregulated in AD and PD datasets using the *Piano* R package (Väremo et al., 2013) to perform gene set enrichment analysis (GSEA) (Mootha et al., 2003; Subramanian et al., 2005), by default on t-statistics, but also on B-statistics of differential gene expression for the AD *Disease* and *Neuronal proportion* effects. We also used the AD cell-type marker genes defined by Kelley et al. (2018) as a gene set. For GSEA on genes commonly changed in AD and PD (Figure 7B), we used −log_10_(FDR) when both AD and PD scores were positive, log_10_(FDR) when both negative, and zero when signs were contradictory.

### Identifying Candidate Compounds for Reverting Disease-Associated Gene Expression Alterations

We used *cTRAP* (de Almeida et al., 2020) to compare the changes in gene expression induced by thousands of drugs in human cell lines, available in the Connectivity Map (CMap) (Subramanian et al., 2017), with those in human brains that we have inferred to be related to the intrinsic (i.e., systemic) AD and PD effects. As input for *cTRAP*, we used the aforementioned scores for the *Disease* and *Neuronal proportion* effects, thereby ranking changes that are coherent between the MayoClinic and Nativio datasets for AD and the Dumitriu and Zhang datasets for PD, as well as those coherent between AD and PD. The compounds, in clinical trials or launched, with their perturbation *z*-scores (Subramanian et al., 2017) exhibiting the 20 most negative and the 20 most positive average (across different cell lines) Spearman’s correlation with the *Disease* effect scores across common genes, and with an average absolute Spearman’s correlation with the *Neuronal proportion* effect scores <0.05 (to avoid confounding between effects), were selected for AD (Supplementary Figure 15A) and PD (Supplementary Figure 15B) as the top candidates for reversal or induction of disease-associated gene expression alterations for discussion. Noteworthily, cMap includes data for the same compounds tested with different concentrations and at different time points, as well as run in different plate types (ASG, CPD, HOG, etc.) (Supplementary Tables 16–18).

## Results

### The Cellular Composition of AD Brains Is Altered

**FIGURE 1:**
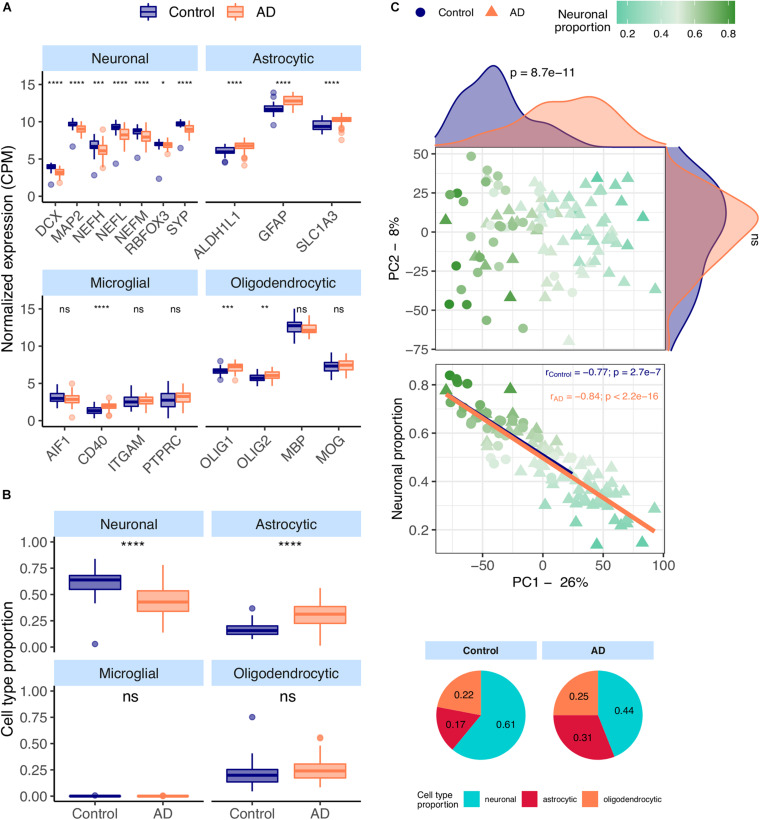
**(A)** Neuronal, astrocytic, microglial, and oligodendrocytic known markers’ expression in the MayoClinic samples. *T*-tests were used to compare gene expression mean differences between diseased (AD) and non-diseased (Control) samples. **(B)** Estimates of the composition of MayoClinic samples in each main cell type based on the human cell type gene expression signature. Wilcoxon signed-rank tests were used to compare differences in proportions between diseased (AD) and non-diseased (Control) samples. **(C)** Sample factorial map (upper plot) of components 1 (PC1) and 2 (PC2) of Principal Component Analysis (PCA) of the gene expression in MayoClinic samples, and their neuronal proportion related to PC1 loadings (lower plot). Indicated in the respective axes’ labels are the percentages of data variance explained by PC1 and PC2. Kolmogorov-Smirnov tests were used to compare the distributions of PC1 and PC2 loadings between AD and Control samples, illustrated by the smoothed histograms along the respective axes of the PCA plot. In the lower plots, the colored solid lines represent the linear regressions between neuronal proportions and PC1 loadings for AD and Control Samples. The respective Pearson’s correlation coefficients (*r*) and associated significance (*p*) are also indicated. Legend: ns: non-significant, ****p ≤ 0.0001, ***p ≤ 0.001, **p ≤ 0.01, *p ≤ 0.05.

Most neuronal markers [*DCX* (Tanapat, 2013), *MAP2* (Tanapat, 2013), *NEFM* (Tanapat, 2013), *NEFH* (Tanapat, 2013), *NEFL* (Tanapat, 2013), *RBFOX3* (Tanapat, 2013), *SYP* (Tanapat, 2013)] are significantly downregulated in AD temporal cortex samples from the MayoClinic dataset (Figure 1A). In contrast, all astrocytic [*ALDH1L1* (Preston et al., 2019), *GFAP* (Preston et al., 2019)), *SLC1A3* (Preston et al., 2019)] and a few microglial and oligodendrocytic markers [*CD40* (Ponomarev et al., 2006), *OLIG1* (Ligon et al., 2004) and *OLIG2* (Ligon et al., 2004)] are significantly upregulated in AD brains.

CIBERSORTx (Newman et al., 2019), a tool that estimates cell type abundances in tissues from their bulk transcriptomes and machine learning-inferred cell-type-specific gene expression profiles, was used to derive the composition in major cell types (astrocytes, microglia, neurons and oligodendrocytes) of AD brain samples (see section “Materials and Methods”). These estimates (Figure 1B) are concordant with the observations in Figure 1A, including significant increase and decrease, respectively, in the proportions of astrocytes and neurons in AD brain samples. Despite the known differences in gene expression between mouse and human brain cells (Hodge et al., 2019), the same trends can be seen using the mouse signature (Supplementary Figures 9A,B). We also performed principal component analysis (PCA) on normalized gene expression in the MayoClinic brain samples. The neuronal composition, along with the disease effect, is correlated with the first principal component (PC1), i.e., that retaining the most data variance (Figure 1C – rho = −0.88; *p* < 2.2e-16). Sex also shows a strong association with PC1 (Supplementary Figure 9E) but there is no significant difference in age or neuronal proportion between female and male individuals (Supplementary Figure 9F).

### AD Alters Cortical Gene Expression Independently From Neuronal Loss

**FIGURE 2:**
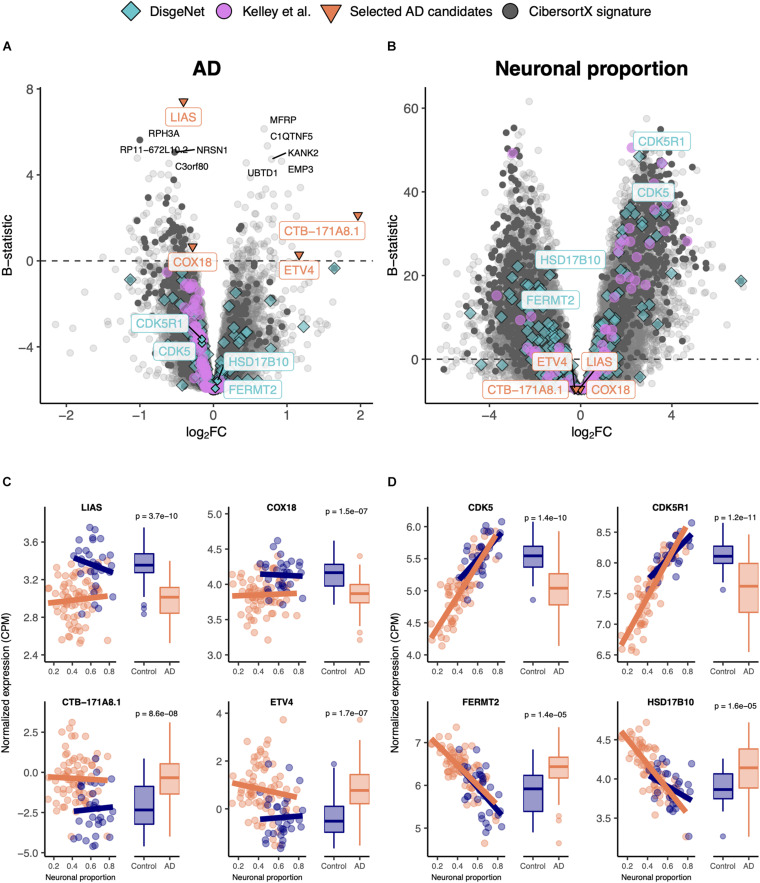
**(A,B)** Volcano plots, relating log_2_ fold-changes (log_2_FC) and B-statistics, of differential gene expression associated with the **(A)** AD and **(B)** Neuronal proportion effects in MayoClinic samples. Highlighted with larger colored dots are disease-associated genes from DisgeNet (Piñero et al., 2019) (light blue), genes reported by Kelley et al. (2018) as undergoing cell-type-specific changes in AD (pink), manually selected gene candidates for AD-specific alterations (orange), and genes included in the CIBERSORTx-derived expression signature for the major brain cell types (dark gray). Amongst all these, labeled are genes of particular interest, with individual expression profiles plotted in panels **(C,D)**. The other labeled genes are the top 10 differentially expressed genes. **(C)** Expression of manually selected gene candidates for AD-specific alterations (labeled in orange in panels **(A,B**) in Control and AD samples – scatterplots against neuronal proportion on the left, boxplots of distribution by condition on the right. **(D)** Same as **(C)** for selected DisgeNet genes (labeled in light blue in panels **A,B)**. Colored solid lines in panels **C,D**) represent linear regressions. *T*-tests, for which *p*-values are indicated, were used to compare gene expression mean differences between Control and AD samples.

We linearly modeled gene expression in the MayoClinic brain samples as a function of technical (RIN) and biological variables, such as neuronal proportion (reflecting neuronal loss), systemic AD, Age, Sex and interaction between neuronal proportion and AD (Supplementary Table 6, Figure 2, and Supplementary Figure 10A). We were thereby able to discriminate genes whose expression is significantly systemically affected by AD (Figures 2A,B) from those essentially showing a strong association with neuronal loss (Figures 2C,D). For instance, *LIAS*, *CTB-171A8.1*, *COX18*, and *ETV4* exemplify genes that show a strong intrinsic AD effect, independent of neuronal proportion (Figure 2B). Moreover, genes that have previously been reported as playing a role in AD in the DisGeNet database, namely *CDK5* (Liu et al., 2016), *CDK5R1* (Moncini et al., 2017), *FERMT2* (Shulman et al., 2014), and *HSD17B10* (Marques et al., 2009), were actually found to be associated with neuronal loss rather than with the disease component (Figure 2D). Additionally, with the *Interaction* effect we were also able to detect genes, such as *PNPLA5* and *PTPN20A* (Supplementary Figure 10C), whose expression was differentially altered with cellular composition in AD brains compared with non-AD samples. It is worth noting that AD genes specific of a cell type, as defined by Kelley et al. (2018), are also more related with the neuronal composition effect (p_GSEA_ = 0.0001) than with the disease effect (p_GSEA_ = 0.6) (Figures 2A,C). Additionally, most up-regulated AD-specific genes seem to be related with cell survival and immune pathways, whereas down-regulated ones with oxidative phosphorylation and Parkinson’s disease (Supplementary Figure 10C).

### AD-Specific Genes are Validated in an Independent Dataset

**FIGURE 3:**
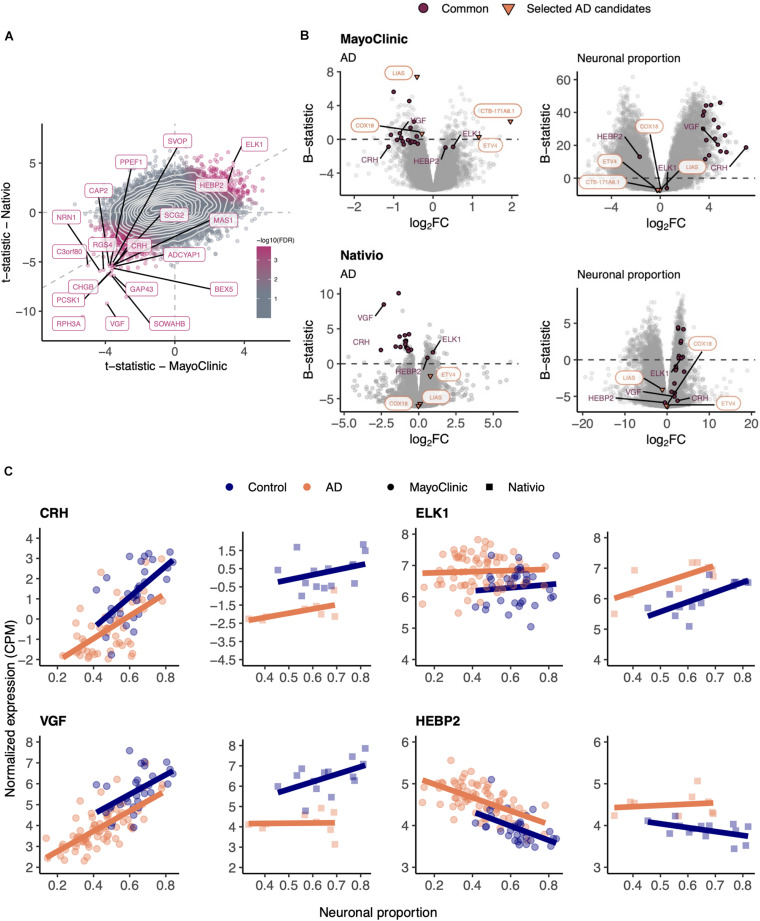
**(A)** Scatter plot comparing the *t*-statistics of differential gene expression associated with the AD effect in MayoClinic and Nativio samples. Points (genes) are colored according to the FDR of the random permutation test on the product of the *t*-statistics (section “Materials and Methods”). Labeled genes are those significantly differentially expressed (FDR < 0.05) in both datasets and significant in that permutation test (FDR < 0.05). Light gray dashed zero and identity lines, light gray solid contour density lines. **(B)** Volcano plots, relating log_2_ fold-changes (log_2_FC) and B-statistics, of differential gene expression associated with the of AD and Neuronal proportion effects in MayoClinic and Nativio samples. Genes highlighted in orange are those manually selected as candidates for AD-specific alterations already represented in Figure 2, those labeled in panel **(A)** are here highlighted in purple (“Common”), and those here labeled in purple are presented in panel **(C)**. **(C)** Expression of manually selected “Common” genes in MayoClinic and Nativio samples. Colored solid lines represent linear regressions.

In order to validate those results, we used the independent AD RNA-seq dataset (lateral temporal lobe) (Nativio et al., 2018), herein named Nativio (Table 1), that we found to better match the larger MayoClinic dataset (temporal cortex) (Allen et al., 2016) in terms of brain area. Although the Nativio dataset did not present significant differences in cellular composition between AD and non-AD samples (Supplementary Figure 11A), its samples were from significantly younger donors (*p* = 2.7e-10) and the neuronal proportion of its AD samples is significantly different (*p* = 0.033) from MayoClinic AD samples’ (Supplementary Figure 11C), we found consistency in AD-associated gene expression changes between the MayoClinic and the Nativio datasets (Figure 3 and Supplementary Tables 7–9).

### The Human Brain Cellular Composition Is Not Significantly Altered by PD

**FIGURE 4:**
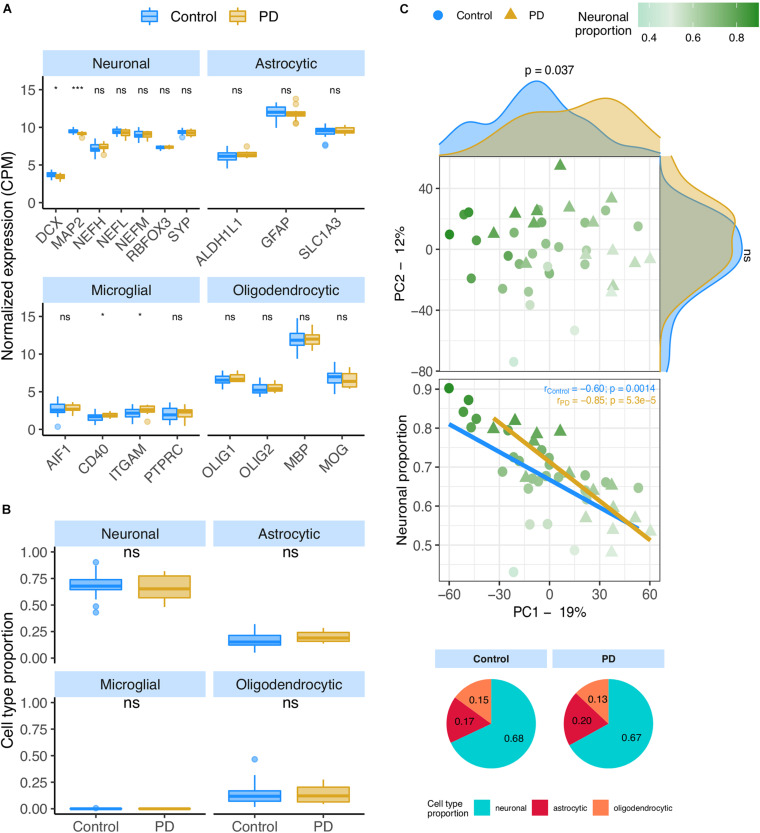
**(A)** Neuronal, astrocytic, microglial, and oligodendrocytic known markers’ expression in the Dumitriu samples. *T*-tests were used to compare gene expression mean differences between diseased (PD) and non-diseased (Control) samples. **(B)** Estimates of the composition of Dumitriu samples in each main cell type based on the human cell type gene expression signature. Wilcoxon signed-rank tests were used to compare differences in proportions between diseased (PD) and non-diseased (Control) samples. **(C)** Sample factorial map (upper plot) of components 1 (PC1) and 2 (PC2) of Principal Component Analysis (PCA) of the gene expression in Dumitriu samples, and their neuronal proportion related to PC1 loadings (lower plot). Indicated in the respective axes’ labels are the percentages of data variance explained by PC1 and PC2. Kolmogorov-Smirnov tests were used to compare the distributions of PC1 and PC2 loadings between PD and Control samples, illustrated by the smoothed histograms along the respective axes of the PCA plot. In the lower plots, the colored solid lines represent the linear regressions between neuronal proportions and PC1 loadings for PD and Control Samples. The respective Pearson’s correlation coefficients (*r*) and associated significance (*p*) are also indicated. Legend: ns: non-significant, ****p ≤ 0.0001, ***p ≤ 0.001, **p ≤0.01, *p ≤0.05.

We analyzed the PD datasets following similar approaches to those used on AD transcriptomes. In the RNA-seq PD dataset (Dumitriu), only two neural (*DCX* and *MAP2*) and two microglial (*CD40* and *ITGAM*) markers showed significant alterations between PD and non-PD samples (Figure 4A), concordantly with no significant differences in cellular composition as estimated by CIBERSORTx (Figure 4B) using both the human and the mouse signatures (Supplementary Figures 12A,B). However, the neuronal composition of samples, along with the disease effect, has a significant association with PC1 (Figure 4C – rho = −0.66; *p* = 3.2e-6) of gene expression. Age also shows a relationship with PC1 (Supplementary Figures 12C,E) but no significant age difference exists between PD and non-PD sample donors (Supplementary Figure 12F).

### PD Alters Cortical Gene Expression Independently From Neuronal Loss

**FIGURE 5:**
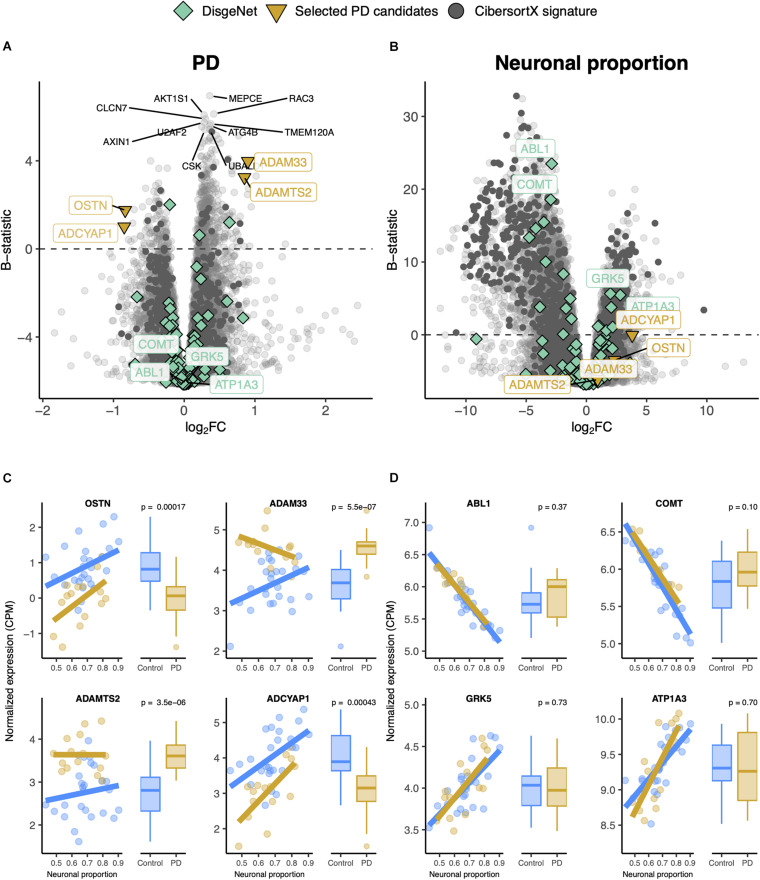
**(A,B)** Volcano plots, relating log_2_ fold-changes (log_2_FC) and B-statistics, of differential gene expression associated with the **(A)** PD and **(B)** Neuronal proportion effects in Dumitriu samples. Highlighted with larger colored dots are disease-associated genes from DisgeNet (Piñero et al., 2019) (light green), manually selected gene candidates for PD-specific alterations (yellow), and genes included in the CIBERSORTx-derived expression signature for the major brain cell types (dark gray). Amongst all these, labeled are genes of particular interest, with individual expression profiles plotted in panels **(C,D)**. The other labeled genes are the top 10 differentially expressed genes. **(C)** Expression of manually selected gene candidates for PD-specific alterations (labeled in yellow in panels **A,B**) in Control and PD samples – scatterplots against neuronal proportion on the left, boxplots of distribution by condition on the right. **(D)** Same as **(C)** for selected DisgeNet genes (labeled in light green in panels **A,B**). Colored solid lines in panels **(C,D)** represent linear regressions. *T*-tests, for which *p*-values are indicated, were used to compare gene expression mean differences between Control and PD samples.

Gene expression was modeled for each gene as a function of the technical variables (RIN and unknown confounder), neuronal proportion (i.e., neuronal loss), intrinsic PD and Age (Figure 5A, Supplementary Figure 13A and Supplementary Table 10). No PD-neuronal loss interaction effect was considered because no significant differences in cell type proportions between PD and non-PD samples were detected (Figure 4B). We were thereby able to discriminate genes with a strong disease effect (Figure 5B) from those essentially altered by neuronal loss (Figure 5D). In fact, according to our analysis, genes reported as playing a role in PD [*ABL1* (Mahul-Mellier et al., 2014), *COMT* (Jiménez-Jiménez et al., 2014), *GRK5* (Liu et al., 2010), and *APT1A3* (Haq et al., 2019)] were found associated with neuronal loss rather than the disease itself (Figures 5A,C).

### PD-Specific Genes are Validated in an Independent Dataset

**FIGURE 6:**
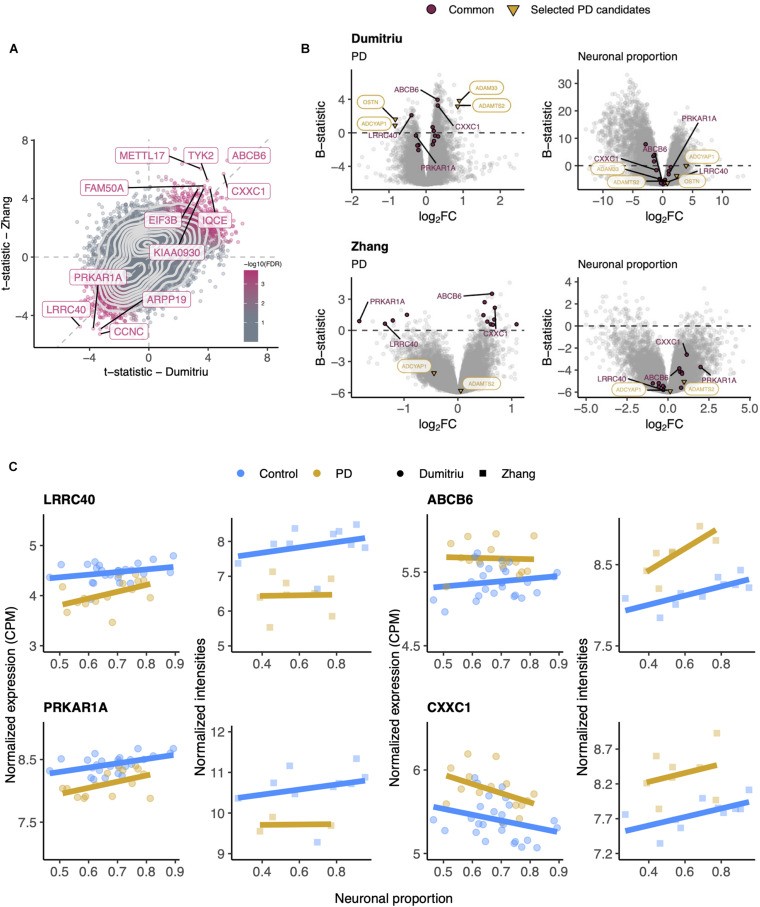
**(A)** Scatter plot comparing the t-statistics of differential gene expression associated with the PD effect in Dumitriu and Zhang samples. Points (genes) are colored according to the FDR of the random permutation test on the product of the *t*-statistics (section “Materials and Methods”). Labeled genes are those significantly differentially expressed (FDR < 0.05 – Dumitriu and FDR < 0.11 – Zhang, section “Materials and Methods”) in both datasets and significant in that permutation test (FDR < 0.05). Light gray dashed zero and identity lines, light gray solid contour density lines. **(B)** Volcano plots, relating log_2_ fold-changes (log_2_FC) and B-statistics, of differential gene expression associated with the of PD and Neuronal proportion effects in Dumitriu and Zhang samples. Genes highlighted in orange are those manually selected as candidates for PD-specific alterations already represented in Figure 5, those labeled in panel **(A)** are here highlighted in purple (“Common”), and those labeled in purple are presented in panel **(C)**. **(C)** Expression of manually selected “Common” genes in Dumitriu and Zhang samples. Colored solid lines represent linear regressions.

Although RNA-seq provides a more precise quantification of gene expression than microarrays (Wang et al., 2009), we could not find any other independent PD RNA-seq dataset matching, in terms of brain region, the Dumitriu study and therefore resorted to the Zhang microarray study. This independent dataset did not present any significant cellular composition alteration between PD and non-PD samples (Supplementary Figure 14A) either. Additionally, we found no significant differences in neuronal proportion estimates or age between samples from the Dumitriu and Zhang studies (Supplementary Figure 14C). We find gene expression changes that are consistent between the two analyzed PD datasets (Figures 6A,B and Supplementary Tables 10–13), including for *LRRC40* and *ABCB6* (Figure 6C). However, from the selected PD candidates as examples shown in Figure 5, only *ADAMTS2* and *ADCYAP1* were profiled in the Zhang dataset and did not recapitulate the changes observed in the Dumitriu dataset (Figure 6B).

### Common AD- and PD-Associated Gene Expression Alterations are Related With Cell Survival and Metabolism

**FIGURE 7:**
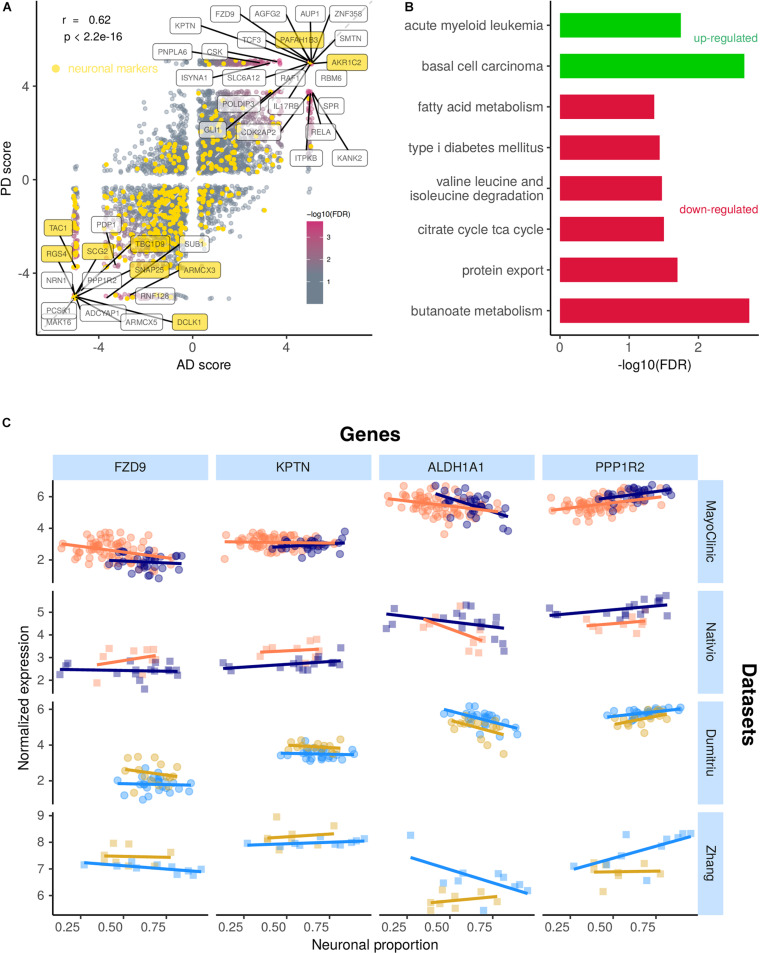
**(A)** Scatter plot comparing the combined scores of differential gene expression (section “Materials and Methods”) associated with the AD and the PD effects. Labeled genes are those highly significant (FDR < 0.0005) in the random permutation test of the product of scores (section “Materials and Methods”). The Pearson’s correlation coefficient (*r*) between scores and associated significance (*p*) are also indicated. Neuronal gene markers included in the CIBERSORTx-derived cell-type expression signature depicted in yellow. Identity line in dashed gray. **(B)** Significance of enrichment of KEGG pathways in genes up-regulated (green) and down-regulated (red) in both AD and PD (section “Materials and Methods”). **(C)** Expression of selected genes commonly altered in AD and PD in samples from all analyzed datasets against their neuronal proportion.

Alzheimer’s diseases and PD-associated gene expression changes in human brains are very correlated (Figure 7A and Supplementary Tables 14, 15), suggesting commonalities in the molecular mechanisms underlying both diseases. Although some neuronal markers are amongst the genes commonly altered by AD and PD, most of them are not, indicating effectivity in decoupling the neuronal loss effect on gene expression (Figure 7A). Genes consistently up-regulated in both diseases are linked to Wnt signaling (basal cell carcinoma pathway) and NF-KB signaling (acute myeloid leukemia) (Figure 7B). Indeed, the genes driving the basal cell carcinoma pathway are *FZD9*, *FZD7*, *FZD2*, *DVL1*, and *AXIN1*, all playing a role in the Wnt signaling pathway, which has already been linked to AD and PD (Harvey and Marchetti, 2014). The genes contributing the most to the acute myeloid leukemia pathway (*RAF1*, *RELA*, and *IKBKB*) are related with NF-KB signaling, a process already known to also play a role in AD and PD (Mattson and Meffert, 2006). Genes consistently down-regulated in both diseases are linked essentially to cell metabolism (Figure 7B). Although the magnitude of disease-induced changes in gene expression is generally modest (as expected, as samples from the same type of tissue are being compared), reassuringly they are overall quite independent from the neuronal composition of the brain samples (Figure 7C).

### Metaraminol Administration Is Inversely Correlated with the AD- and PD-Gene Expression Phenotype

**FIGURE 8:**
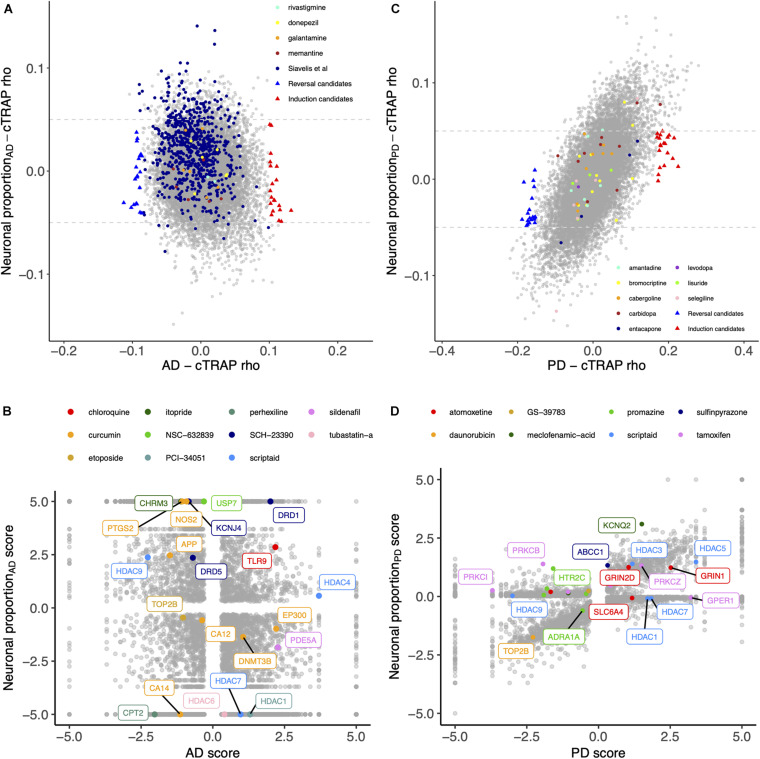
**(A)** Scatter plot comparing, between the AD and the Neuronal proportion effects, the cTRAP-derived cross-gene Spearman’s correlation coefficients (rho) of their differential expression combined scores with perturbation *z*-scores for cMap compounds. Highlighted with blue and red triangles are compounds selected as top candidates for, respectively, reversal and induction of AD-associated gene expression alterations (Supplementary Figure 15A; section “Materials and Methods”). Colored circles highlight compounds in use for AD treatments, including those listed by Siavelis et al. (2016). **(B)** Scatter plot comparing the combined scores of differential gene expression between the AD and the Neuronal proportion effects. Highlighted genes are known targets of selected candidate compounds for reversal of AD-associated gene expression alterations (Supplementary Figure 15A). **(C)** Scatter plot comparing, between the PD and the Neuronal proportion effects, the cTRAP-derived cross-gene Spearman’s correlation coefficients (rho) of their differential expression combined scores with perturbation *z*-scores for cMap compounds. Highlighted with blue and red triangles are compounds selected as top candidates for, respectively, reversal and induction of PD-associated gene expression alterations (Supplementary Figure 15B; section “Materials and Methods”). Colored circles highlight compounds in use for PD treatments. **(D)** Scatter plot comparing the combined scores of differential gene expression between the PD and the Neuronal proportion effects. Highlighted genes are known targets of selected candidate compounds for reversal of PD-associated gene expression alterations (Supplementary Figure 15B).

**FIGURE 9:**
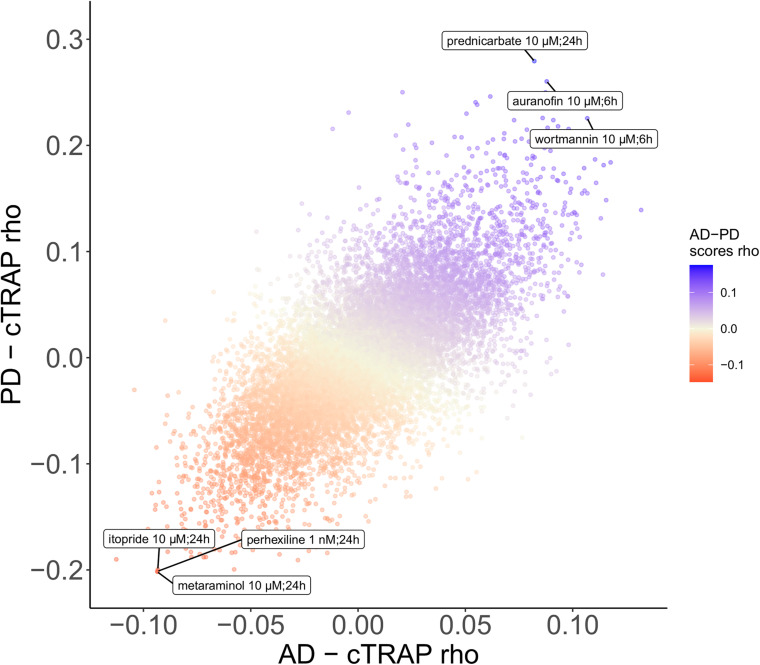
Scatter plot comparing, between the AD and the PD effects, the cTRAP-derived cross-gene Spearman’s correlation coefficients (rho) of their differential expression combined scores with perturbation *z*-scores for cMap compounds. Points (compounds) are colored according to the cross-gene Spearman’s correlation coefficients of their perturbation *z*-scores with the scores for common AD-PD differential expression (section “Materials and Methods”). Labeled compounds are the three most correlated and the three most anti-correlated compounds.

We used cTRAP (de Almeida et al., 2020) to identify drugs that, when delivered to human cell lines, cause similar (correlated) or opposite (anti-correlated) gene expression changes to those we observed as intrinsically associated to AD and PD (Figures 8A,C). Interestingly, gene expression perturbations induced by drugs known to be used in the clinic to treat AD (Siavelis et al., 2016) (donepezil, galantamine, memantine, and rivastigmine) and PD (Zahoor et al., 2018) (amantadine, bromocriptine, cabergoline, carbidopa, entacapone, levodopa, lisuride, and selegiline) were not amongst the most correlated with those by the respective target diseases (Figures 8A,B and Supplementary Tables 16–18). Siavelis et al. (2016) had followed a similar approach, although they did not decouple the neuronal loss effect, having identified 27 drugs linked to the AD phenotype (Figure 8A). Chloroquine and scriptaid seem promising drug candidates for AD since their known targets are indeed overexpressed in AD and vary very little with neuronal loss (Figure 8B). Scriptaid also seems promising for PD therapeutics for similar reasons (Figure 8D). Additionally, gene expression changes upon metaraminol administration showed up as being the most anti-correlated with those commonly induced by AD and PD (Figure 9), being metaraminol therefore a potential candidate drug to be tested for repurposing. Gene expression changes upon wortmannin administration are, in a dose dependent manner, the most correlated with those commonly induced by AD and PD (Figure 9).

## Discussion

In this study, we investigated the impact of cellular composition on Alzheimer’s and Parkinson’s diseases’ molecular effects in human brains. AD and PD brains are characterized by a loss of neurons and an increase of astrocytic reactivity when compared to age-matched healthy brain samples (Gomez-Isla et al., 1997; MacDonald and Halliday, 2002; Verkhratsky et al., 2010; Booth et al., 2017). Signatures of these changes will be confounded with disease-intrinsic molecular alterations, both systemic and cell type-specific, in any differential expression analysis between diseased and healthy brains. We confirmed this by looking at the expression of known neuronal, astrocytic, oligodendrocytic and microglial markers in AD and PD transcriptomic datasets. We indeed found neuronal and astrocytic markers significantly downregulated and upregulated, respectively, in MayoClinic AD samples compared to controls (Figure 1A). Although microglia were reported to be involved in AD (Solito and Sastre, 2012) and PD (Le et al., 2016), we only observed a significant increase in one (*CD40*) out of four microglial markers tested in AD (Figure 1A) and in two (*CD40* and *ITGAM*) in PD samples (Figure 4A). Since microglia represent a small subset of human brain cells [5 to 15% of human brain cells (Pelvig et al., 2008)], there were likely too few microglial cells in the profiled brain sections for their transcriptomic signal to be properly detected, as suggested by our digital cytometry estimates (Figures 1B, 4B). Still, the detection of a significant increase in *CD40* and *ITGAM* microglial markers in PD samples needs further investigation, as the highest concentration of microglia in the brain is located in the *substantia nigra* (Block et al., 2007), the first region affected by the loss of dopaminergic neurons in PD (Badger et al., 2014). This could then induce a more reactive response of microglia in PD cortices.

Based on the evidence that cellular composition was altered in AD and PD brain samples, we computationally estimated therein the proportion of the main brain cell types: neurons, astrocytes, microglia and oligodendrocytes. We derived a gene expression signature (1 962 genes – Supplementary Table 2) from a publicly available single-cell RNA-seq dataset of human adult cortical samples (Darmanis et al., 2015) to distinguish those four cell types. To test the specificity of those signatures, we used CIBERSORTx (Newman et al., 2019) to estimate the composition of samples from an independent single-cell RNA-seq dataset (Zhang et al., 2016) of human neurons, microglia, astrocytes and oligodendrocytes. Each of these cells was mostly assigned to its respective pre-annotated cell type (Supplementary Figure 7A). Some oligodendrocyte samples showed a small presence of the other three cell types that might be related with the myelin of oligodendrocytes having some debris of astrocytes, microglia and neurons attached, given that oligodendrocytes closely interact with those cells (Domingues et al., 2016). With the further advances in scRNA-seq technologies and the accumulation of human brain single-cell datasets in healthy and diseased conditions (Mathys et al., 2019b), the major brain cell type signatures will be further improved and allow an increase in sensitivity of digital cytometry.

After validating the cell type signatures, we used them to estimate the proportion of neurons, astrocytes, microglia and oligodendrocytes in AD and PD brain samples from their bulk transcriptomes. In line with differences in expression in canonical markers illustrated in Figure 1A, the estimated neuronal proportion was significantly lower in AD compared with control brains (Figure 1B). Some samples reached up to 60–90% of neurons, much higher than estimates based on cell counting (Pelvig et al., 2008; Herculano-Houzel, 2009). This is likely related with the neuronal RNA content being up to 2-fold as much as that of glial cells (Filipchenko et al., 1976). In this study we are therefore estimating the relative contribution by each cell type to the total amount of mRNA in the bulk samples and not their actual proportion of the total number of cells.

Linear models are a commonly used statistical approach to model gene expression as a function of relevant explanatory variables. Here, these were potential technical biases (RIN and an unknown confounder variable), age, sex (for AD datasets), estimated neuronal proportion (neuronal loss), disease (categorical AD or PD) and, for the AD datasets, interaction between neuronal proportion and disease. Considering that AD and PD are age-related neurodegenerative disorders, it is expected that most of their associated gene expression changes in the brain are result from the loss of neurons and aging, therefore the need to estimate their independent effects and decouple them from the intrinsic molecular effects of the diseases that we are interested in. Using those models, we identified genes whose expression was significantly affected by the intrinsic (systemic) disease effect (Figures 2, 5), as well as genes whose expression was mostly explained by the other effects (Supplementary Figures 10A, 13A). However, we were not able to completely decouple the explanatory variables, as the associated moderated t statistics of differential expression were to some extent correlated with each other (Supplementary Figures 10D, 13C). The correlations between RIN and the intrinsic disease and neuronal loss effects may be explained by potential agonal conditions, such as patients being in a coma or their brains undergoing hypoxia just before death, preceding the collection of *post-mortem* samples (Tomita et al., 2004). For AD, with the *Interaction* effect, we were able to detect genes whose expression varies differently upon neuronal loss in AD samples (Supplementary Figure 10C). For example, the *PNPLA5* gene is involved in lipid metabolism (Kim et al., 2016) and is thought to play a role in the autophagy biogenesis (Dupont et al., 2014). Those processes have been implicated in AD (Li et al., 2017; Zarrouk et al., 2018) and a variant in *PNPLA5* was reported to be associated with the *APOE* genotype directly linked to AD (Cruchaga et al., 2012). Another example is *PTPN20A*, encoding a phosphatase with a dynamic subcellular distribution that targets sites of actin polymerization, a fundamental cellular process (Fodero-Tavoletti et al., 2005). Although, to our knowledge, no reports have linked *PTPN20A* to AD, it might indicate that, concomitant with the loss of other neurons, AD neurons suffer more structural changes than non-AD neurons.

The importance of decoupling the intrinsic disease effect from others can be seen in Figures 2B, 5B. Looking at some of the genes already reported as potentially playing a role in AD [e.g., *CDK5* (Liu et al., 2016)] or PD [e.g., *COMT* (Jiménez-Jiménez et al., 2014)], we observed that alterations in their expression were mostly driven by neuronal loss, i.e., by changes in cellular composition, but not so much by an intrinsic cell type-independent disease effect. This suggests that some genes previously reported as candidates for playing a role in AD or PD may be “false positives” for their association with the diseases’ etiology and, given their cell-type specificity, have been found dysregulated due to changes in cellular composition (Kelley et al., 2018).

As shown in Figures 3A, 6A, the results of differential expression analyses are significantly correlated between independent datasets. Although some genes initially selected as candidates for intrinsic disease markers, such as *ETV4* and *LIAS* for AD, were not found to behave consistently in both datasets, others previously described as playing a role in AD and PD, such as *RPH3A* (Tan et al., 2014) and *CXXC1* (Diao et al., 2013), were consistent. We identified genes such as *HEBP2* and *PRKAR1A* to be, respectively, AD and PD-specific (Figures 3C, 6C) and, to our knowledge, they had not been previously linked with the disorders. *HEBP2* is known to play a role in mitochondria and its inhibition has been shown to be important for HeLa cells survival upon oxidative stress (Szigeti et al., 2010). Considering that *HEBP2* is upregulated in our AD samples, its overexpression may contribute to the sudden death of neuronal cells upon the AD-characteristic high oxidative stress environment (Tönnies and Trushina, 2017). Moreover, although *HEBP2* has not yet been linked to AD, its homologs *HEBP1* has been described as potentially playing a role in neurons’ ability to sense cytotoxicity over the course of the disease (Yagensky et al., 2019). When PRKAR1A, the cAMP-dependent protein kinase type I-alpha regulatory subunit, is not working properly, it causes an hyperactivation of PKA signaling and its loss of function has been shown to cause cell death and muscle impairment (Gangoda et al., 2014), two PD-related phenotypes.

Being the two most common neurodegenerative disorders in the world, it has already been suggested that AD and PD could share a common mechanism of neurodegeneration (Calne et al., 1986; Xie et al., 2014). For instance, Greenfield and Vaux (2002) proposed that the common mechanism may be associated with the aberrant activation of a developmental process involving a non-classical, non-enzymatic action of acetylcholinesterase. Our results suggest that the genes whose expression is commonly altered in AD and PD are essentially related with cell metabolism and NF-KB and Wnt signaling pathways (Figure 7B), which were already reported as playing a role in PD (Mattson and Meffert, 2006; Cai et al., 2012; Harvey and Marchetti, 2014; Seo and Park, 2019). Another study, that used mice with a deletion of the vesicular acetylcholine transporter in the forebrain, suggests that cholinergic failure causes changes in RNA metabolism that can facilitate Alzheimer’s-like murine pathology (Kolisnyk et al., 2016). Oxidative phosphorylation and Parkinson’s disease pathways were significantly altered in both diseases but enriched in genes downregulated in the MayoClinic AD samples (Supplementary Figure 10B) and upregulated in Dumitriu PD samples (Supplementary Figure 13B). The putative upregulation of the oxidative phosphorylation pathway in PD is mostly driven by NADH dehydrogenase genes such as *NDUFS8*, *NDUFS7*, and *NDUFA11*, which take part in mitochondria’s complex I, already reported to be impaired in PD (Keeney, 2006). Oxidative phosphorylation’s apparent downregulation in AD is mostly driven by *COX* genes such as *COX11* and *COX15*. The most consistent defect in mitochondrial electron transport enzymes in AD is indeed a deficiency in COX (Kish et al., 1992), mitochondria complex IV (Kish et al., 1992). Genes highlighted in Figure 7A should also be considered as candidate targets for functional manipulation in both AD- and PD-related studies, since they may unveil mechanisms that are disrupted in similar ways in both disorders.

Drug discovery for human diseases is a slow and costly process (Insa, 2013), drug repurposing being therefore seen as a faster, safer and cheaper alternative (Siavelis et al., 2016). Using *cTRAP* (de Almeida et al., 2020), we identified drugs, already in clinical trials or launched, that potentially induce gene expression changes that are significantly anti-correlated with those caused by AD and PD (Figures 8A,C). For AD, we identified compounds already linked with the disease. For instance, chloroquine, an antimalarial drug, was shown to increase tau proteolysis (Zhang et al., 2009) as well as to be neuroprotective upon brain injury by diminishing inflammation and neuronal autophagic death (Cui et al., 2015). Tubastatin-a, an HDAC6 inhibitor, was used in AD mice leading to alleviated behavioral deficits, alterations on amyloid-beta load and reduced tau phosphorylation (Zhang et al., 2014). Sildenafil, usually used to treat erectile dysfunction, is currently being investigated in AD therapeutics (Sanders, 2020). Amisulpride and citalopram, two antipsychotic drugs, have been used in AD (Mauri et al., 2006; Porsteinsson et al., 2014). Curcumin has been implicated in AD therapeutics, apparently decreasing beta-amyloid plaques as well as slowing neurodegeneration and acting as an anti-inflammatory (Chen et al., 2018). Doxycycline is a compound known to cross the blood-brain barrier and a very promising candidate since it reduced amyloid-beta oligomers and neuroinflammation in AD mouse models (Balducci and Forloni, 2019). Etoposide needs to be further explored, given a study reporting it as an inducer of cellular senescence and mitochondrial dysfunction in cultured rat astrocytes (Bang et al., 2019) but knowing that rat cell lines may not recapitulate all the molecular cues of the human brain microenvironment. To our knowledge, no research has been reported on the use of interesting candidates panobinostat, dimenhydrinate and perhexiline in AD. Indeed, perhexiline is involved in the inhibition of mTOR pathway which is related with autophagy, a process known to be altered in AD (Jaeger and Wyss-Coray, 2010), and panobinostat acts as an HDAC inhibitor, leading to the hypothesis that it may play a role similar to that of tubastatin-a. For PD, we also identified compounds previously linked to the disease. Atomoxetine, an inhibitor of the norepinephrine reuptake, has been studied in PD therapeutics since the noradrenergic system is involved in executive functions impaired in PD (Warner et al., 2018). Meclofenamic acid, a non-steroid anti-inflammatory drug, has been shown to have an anti-fibrillogenic effect on alpha-synuclein fibrils *in vitro* (Hirohata et al., 2008). Tamoxifen, an estrogen modulator, has also been related with PD treatment but is associated with controversial findings. Although tamoxifen demonstrated neuroprotective effects in some animal and *in vitro* studies (Lee et al., 2009; Mosquera et al., 2014), it has been shown in some cohorts of female breast cancer patients that its usage may increase PD risk (Latourelle et al., 2010; Hong et al., 2017). However, given that our PD analyses were performed only in male samples, our results could suggest a sex-specific mode of action of tamoxifen in PD. Additionally, myricetin has neuroprotective effects in different PD *Drosophila* and rat models (Dhanraj et al., 2018; Huang et al., 2018). To our knowledge, other drugs such as genipin and praziquantel have not yet been related to PD and could be interesting to further explore for repurposing in that context. For instance, genipin is the main component of a Chinese medicinal herb and was shown to have anti-inflammatory and neuroprotective effects that could be beneficial for neurogenerative diseases such as PD (Li et al., 2016). Praziquantel, an anthelmintic compound, could be a very interesting candidate since niclosamide, another anthelmintic drug, has been suggested to be beneficial in PD through the activation of the PINK1 pathway that is usually impaired in PD (Barini et al., 2018).

Metaraminol, an adrenergic agonist that also stimulates the release of norepinephrine and primarily used as a vasoconstrictor in the treatment of hypotension (National Center for Biotechnology Information, 2020. PubChem Database.), induces the gene expression changes most anti-correlated with those by both AD and PD (Figure 9). To our knowledge, there is no association between metaraminol and AD and PD therapeutics. However, adrenergic agonists can decrease noradrenergic degeneration, a characteristic condition of AD patients (Gannon et al., 2015). As for PD, using adrenergic agonists along with levodopa treatment has been shown to lead to a diminishment in parkinsonian symptoms (Alexander et al., 1994). Perhexiline can act as an inhibitor of mTORC1, a protein kinase involved in autophagy, and is able to stimulate autophagy (Balgi et al., 2009). One common shared feature between AD and PD is indeed autophagy decrease (Fujikake et al., 2018), which might explain the anti-correlation between its transcriptomic impact and the expression profiles changes induced by AD and PD (Figure 9). Itopride, a dopamine D2 antagonist with acetylcholinesterase inhibitory actions (National Center for Biotechnology Information, 2020. PubChem Database.), has already been studied as a potential drug for AD given its very similar structure to curcumin, shown to decrease the accumulation of Aß aggregates (Ngo et al., 2016). Moreover, it is also used for increasing gastrointestinal motility, a symptom that is prominent in PD patients, although it also seems to induce parkinsonism (Shin and Chung, 2012). We also found scriptaid (Figures 8A,C), a histone deacetylase (HDAC) inhibitor. HDAC enzymes have already been linked to neurodegenerative diseases and there are already several applications of HDAC inhibitors being tested in such context (Gupta et al., 2020). Interestingly, gene expression changes induced by wortmannin, auranofin and prednicarbate, were the most correlated with those by AD and PD. Indeed, wortmannin has been shown to increase Alzheimer-like tau phosphorylation *in vivo* (Liu and Wang, 2002; Xu et al., 2005) and to diminish the effect of an anti-apoptotic compound in an *in vitro* PD model (Limboonreung et al., 2020). Auranofin, a drug used as an antirheumatic agent, has indeed been linked with AD and PD, but not as an inducer of both disorders (Roder and Thomson, 2015). This result needs to be further explored as, for instance, auranofin seems to act through glial cells but does not stop cytokines secretion from astrocytes (Madeira et al., 2013, 2014). Additionally, these findings result from work in cell lines (Madeira et al., 2014) and mice (Madeira et al., 2013), models that do not recapitulate all the molecular cues of the human brain microenvironment. To our knowledge, there is no association between prednicarbate, a corticosteroid drug with an anti-inflammatory action, and AD and PD therapeutics besides the recommendation of not being used together with memantine, one of the few FDA approved drugs for AD, since it inhibits its action (Wishart et al., 2018). These results show the potential of using *in silico* tools to find existing drugs that could be tested as candidates for the treatment of neurodegenerative diseases.

To our knowledge, this is the first study that decouples the effects of cellular composition, aging and sex from the intrinsic disease effect of AD and PD on gene expression in human brains. However, our study has limitations. We focused on the four major brain cell types but our approach is not sensitive enough to estimate the relative amount of mRNA contributed by microglia, therefore missing the transcriptomic signal of their physiology. Moreover, although we validated our results using independent public datasets, an additional local experimental validation is not feasible due to extreme difficulty in having access to human samples that would be suitable independent replicates of those used to generate the analyzed datasets. Additionally, drugs currently used for AD and PD treatment were not among those our analysis deemed more likely able to revert the AD-/PD-specific gene expression changes. This likely reflects the differences between gene expression changes induced by drugs in cancer cell lines [i.e., those available in CMap (Subramanian et al., 2017), on which *cTRAP* (de Almeida et al., 2020) relies] and those the same drugs would induce in brain cells.

We expect the permanent development of single-cell technologies to help increase the resolution of our understanding of the nuances in each human brain cell type, as well as which molecular perturbations therein are critical to the onset and progression of neurodegenerative diseases such as AD and PD. In fact, there are already some studies using single-cell RNA-seq to characterize the cellular composition in normal brains (Darmanis et al., 2015; Lake et al., 2016; Hagenauer et al., 2017), in neurogenesis and somatic reprogramming to neurons (Shin et al., 2015; Treutlein et al., 2016), as well as in AD brains (Grubman et al., 2019; Mathys et al., 2019a). Nevertheless, as single-cell data are still accumulating and there are several bulk transcriptomes available for brains affected by neurodegenerative disorders, approaches like ours could help in the meantime to unveil some of cellular and molecular complexity associated with neurodegeneration in humans.

In summary, our results show the relevance of modeling and accounting for cell type composition when analyzing molecular alterations associated with neurodegenerative disorders, thereby helping to identify candidate gene targets that are related with the disease itself rather than the consequent loss of neurons. They also illustrate the interest of performing *in silico* analysis of chemical perturbagens as preliminary screens for drug repurposing, helping to find new, more effective drug therapies that could mitigate, or even reverse, some of those neurodegenerative disorders’ phenotypes.

## Data Availability Statement

Publicly available datasets were analyzed in this study. This data can be found here: Darmanis dataset – GEO GSE67835 https://www.ncbi.nlm.nih.gov/geo/query/acc.cgi?acc=GSE67835; Mouse dataset – http://mousebrain.org/downloads.html; Zhang dataset – GEO GSE73721 https://www.ncbi.nlm.nih.gov/geo/query/acc.cgi?acc=GSE73721; MayoClinic dataset – https://www.synapse.org/#!Synapse:syn3163039; Nativio dataset – GEO GSE104704 https://www.ncbi.nlm.nih.gov/geo/query/acc.cgi?acc=GSE104704; Dumitriu dataset – GEO GSE68719 https://www.ncbi.nlm.nih.gov/geo/query/acc.cgi?acc=GSE68719; Zhang dataset – GEO GSE20168 https://www.ncbi.nlm.nih.gov/geo/query/acc.cgi?acc=GSE20168.

## Author Contributions

Both authors contributed to the design and implementation of the research, to the analysis of the results and to the writing of the manuscript.

## Conflict of Interest

The authors declare that the research was conducted in the absence of any commercial or financial relationships that could be construed as a potential conflict of interest.
