# Supplementary material for: Unraveling Targetable Systemic and Cell-Type-Specific Molecular Phenotypes of Alzheimer’s and Parkinson’s Brains With Digital Cytometry
Source: Front Neurosci. 2020 Dec 9;14:607215. doi: 10.3389/fnins.2020.607215 (PMC7756021; doi:10.3389/fnins.2020.607215)
Supplement: Supplementary file 1 [file Data_Sheet_1.PDF]

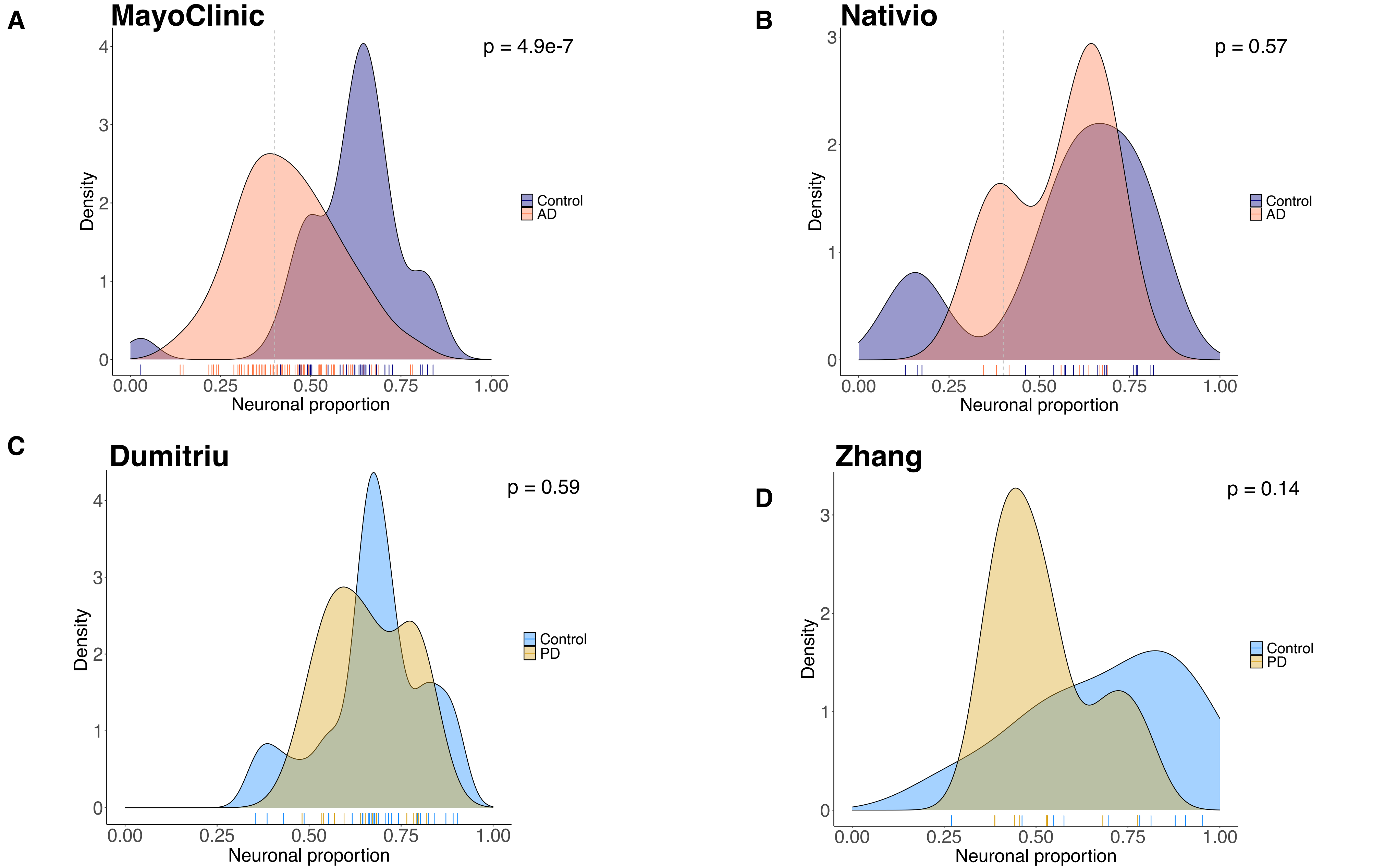

Figure S1 - Smoothed histograms of distributions of neuronal proportions of diseased (AD/PD) and non-diseased (Control) samples from the **(A)** MayoClinic, **(B**) Nativio, **(C)** Dumitriu, and **(D)** Zhang datasets. One MayoClinic and three Nativio Control samples were removed from further analyses due to their very low (< 0.4, vertical dashed lines) neuronal proportions. The significances (p) of Wilcoxon signed-rank tests used to compare differences in proportions between diseased (AD/PD) and non-diseased (Control) samples are shown.

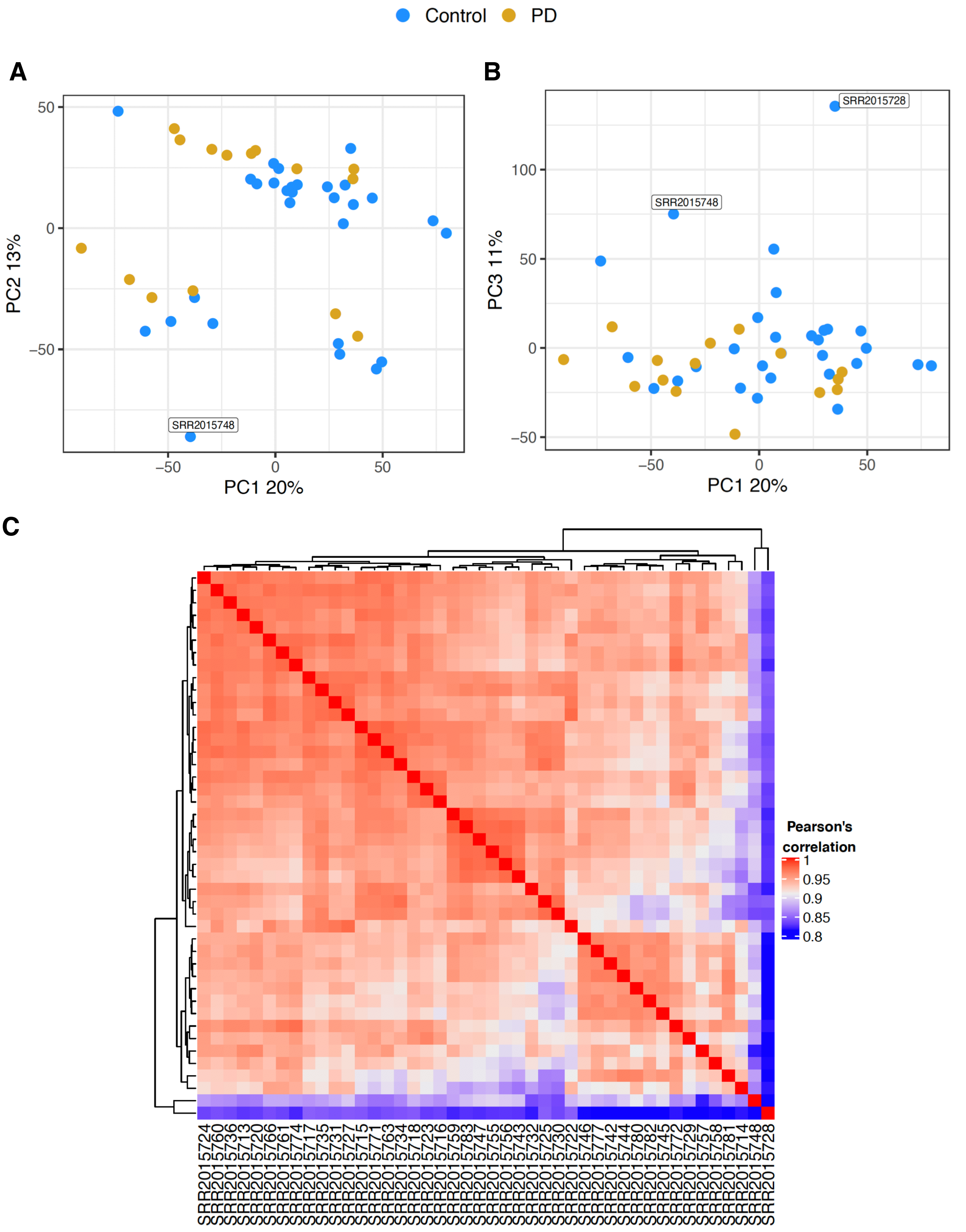

Figure S2 - Sample factorial maps of components **(A)** 1 (PC1) and 2 (PC2), and **(B)** 1 and 3 (PC3), of Principal Component Analysis (PCA) of the gene expression in Dumitriu samples. Indicated in the respective axes labels are the percentages of data variance explained by the components. Labelled are the two samples deemed as outliers and removed from further analyses. **(C)** Heatmap of gene expression correlation between Dumitriu samples, with associated hierarchical clustering, confirming the outlying behavior of the two excluded samples.

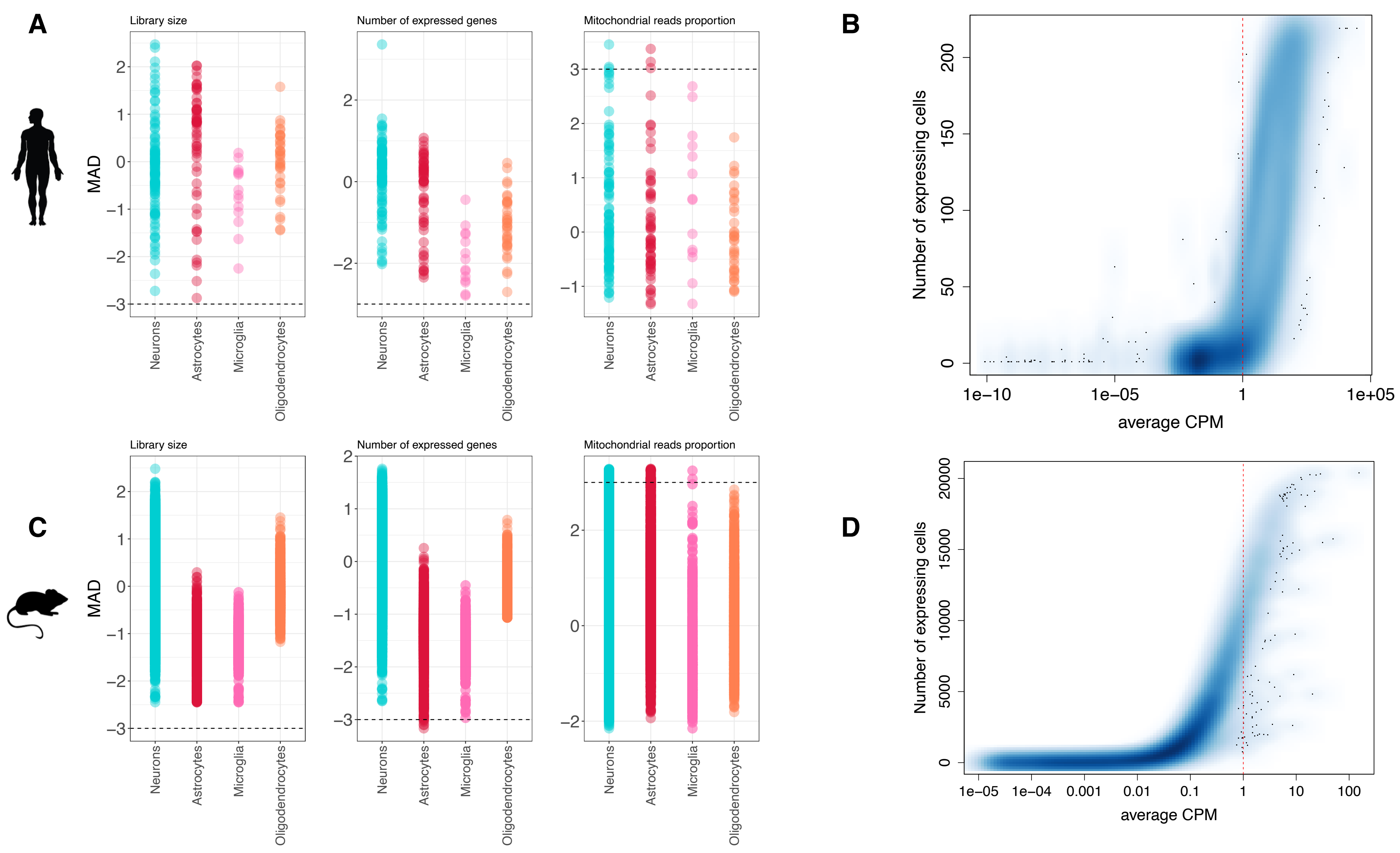

Figure S3 - Dot plots of median absolute deviations (MADs) of library size (i.e. total number of RNA-seq reads), gene counts (i.e. number of genes detected) and proportion of mitochondrial reads (i.e. RNA-seq reads of transcripts from mitochondrial genes) of cell samples from the **(A)** Darmanis and **(C)** Mouse datasets. Cells with MADs below -3 for either library size or gene counts or above 3 for mitochondrial proportion (horizontal black dashed lines) were removed from subsequent analyses.

Smoothed scatter plots relating, for each gene, its average expression across cells with the number of cells in which its expression was detected for the **(B)** Darmanis and **(D)** Mouse datasets. Only genes with average expression higher than 1 CPM (vertical red dashed lines) were kept for subsequent analyses.

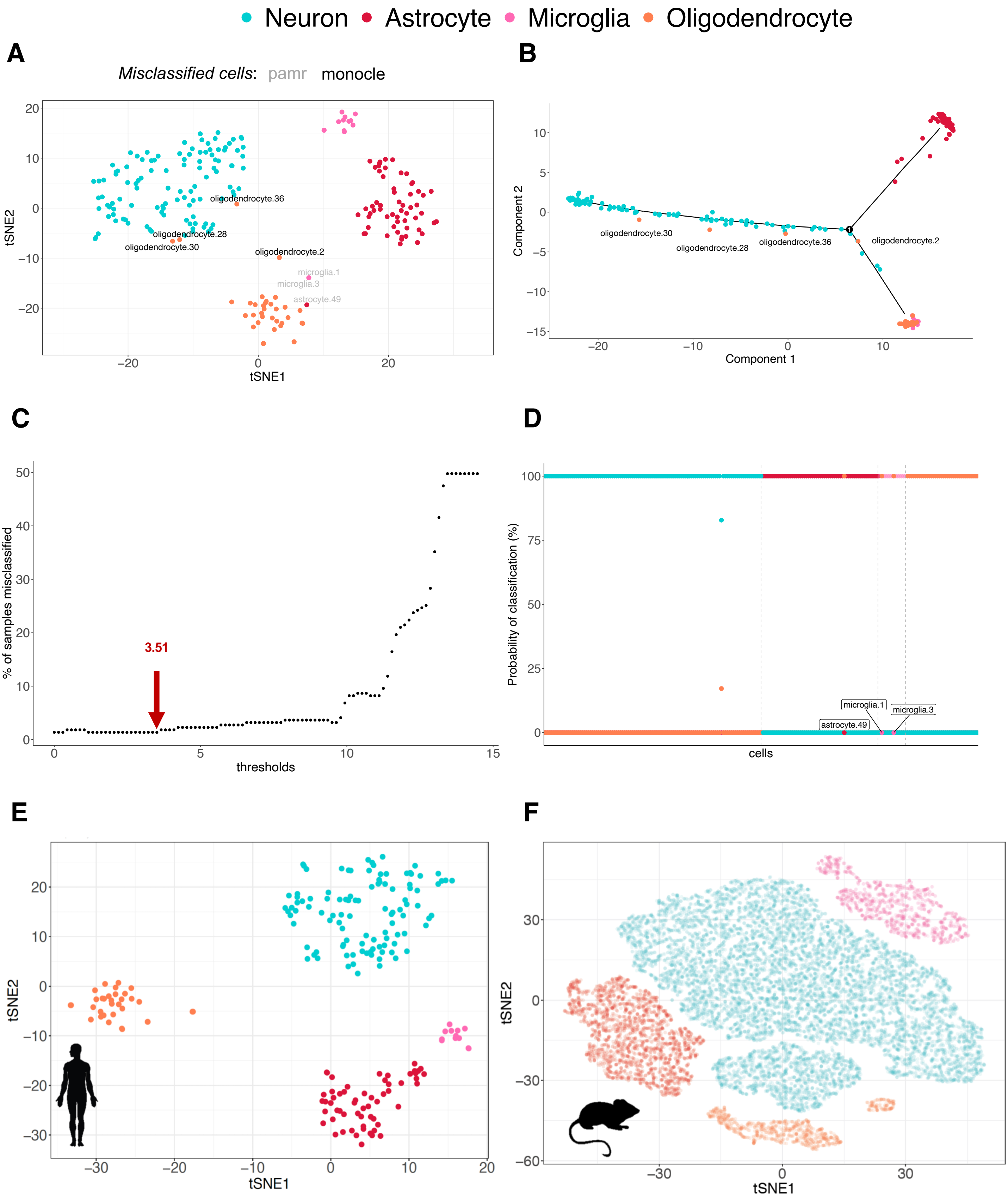

Figure S4 - (A) t-Distributed Stochastic Neighbor Embedding (tSNE) plot of gene expression of Darmanis brain cells. Labelled samples do not cluster with those of their respective annotated cell types, according to *pamr* (gray) or *monocle* (black). **(B)** *monocle*-derived single-cell trajectory analysis in the Darmanis dataset. Labelled are cells that do not map to the same states as those of their annotated cell types. **(C)** Percentage of samples misclassified by *pamr* across centroid shrinkage tresholds. 3.51 was selected, amongst the thresholds with the lowest misclassification error, as that yielding a classifier simultaneously with fewer genes and lower false discovery rate. **(D)** Probability of classification by *pamr* of each Darmanis cell sample in each of the four main brain cell types. Labelled are misclassified cells. **(E)** tSNE plot of gene expression of Darmanis cells without the misclassified samples. **(F)** tSNE plot of gene expression of Mouse cells used to derive the murine cell type signature.

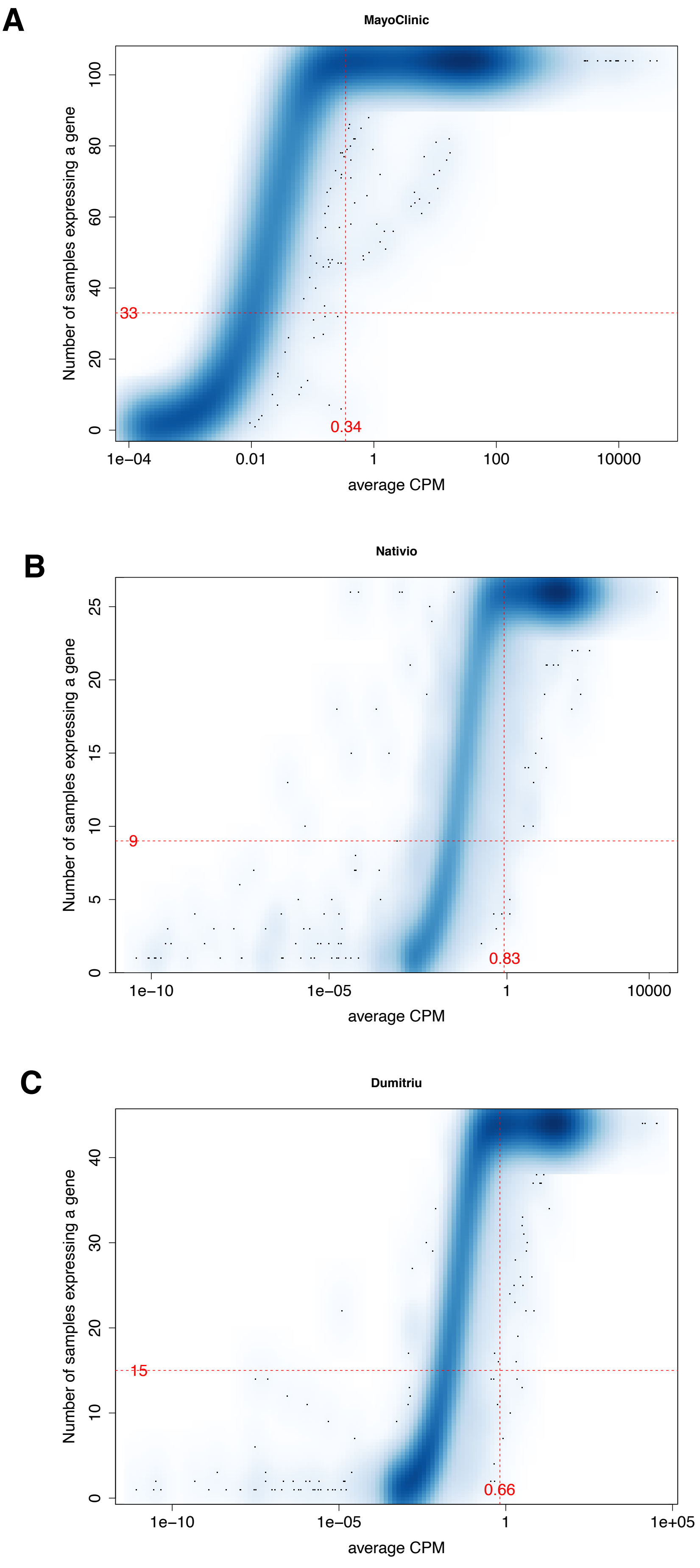

Figure S5 - Smoothed scatter plots relating, for each gene, its average expression across samples with the number of samples in which its expression was detected for the **(A)** MayoClinic, **(B)** Nativio, and **(C)** Dumitriu datasets. Genes kept for analysis have an average expression of at least 0.34, 0.83, and 0.66 CPM and are expressed in at least 33, 9, and 15 samples (red dashed lines) respectively for the MayoClinic, Nativio, and Dumitriu datasets.

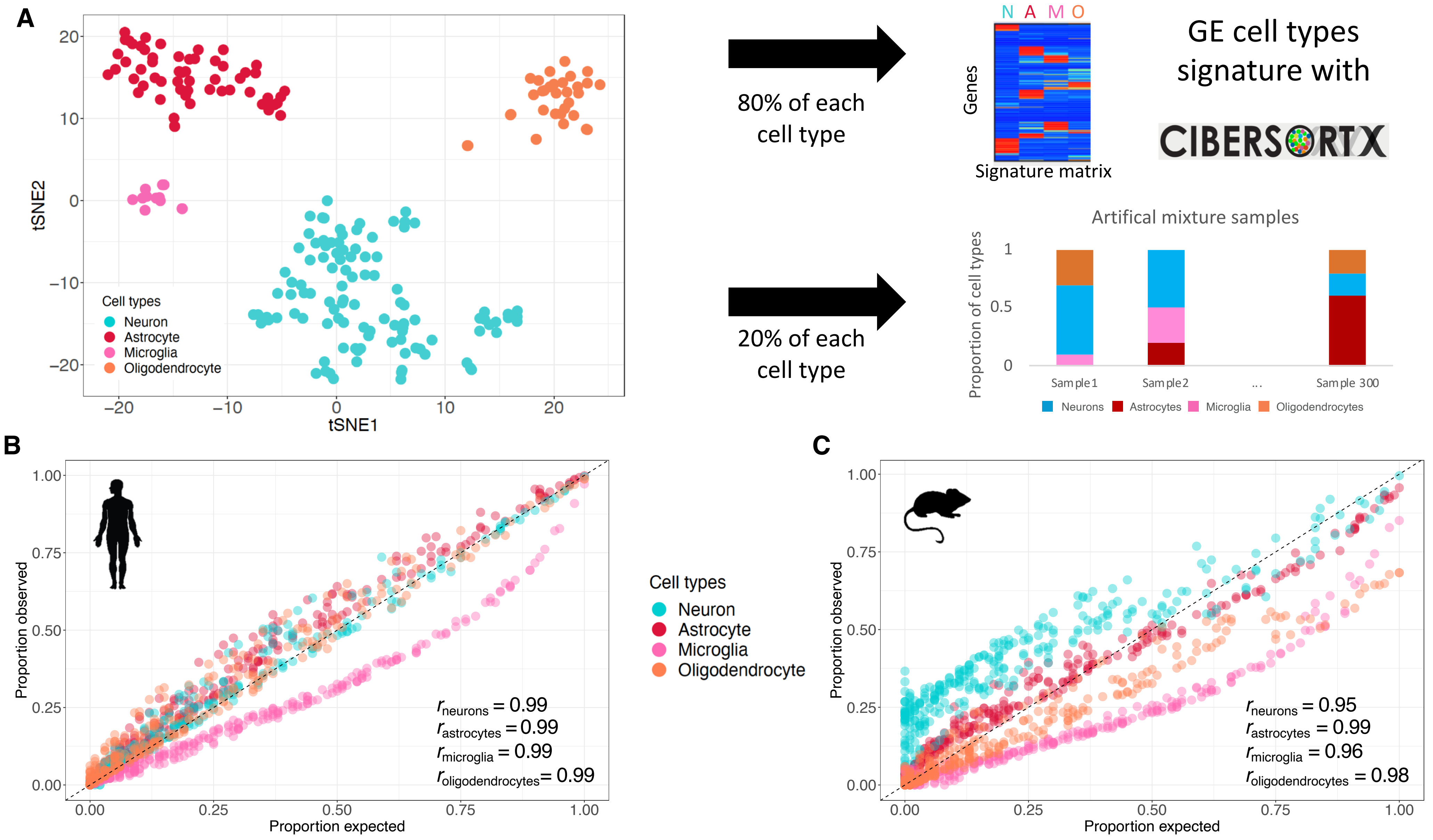

Figure S6 - (A) Pipeline to derive the human cell type signature and the artificial mixture samples from the Darmanis single-cell dataset. CIBERSORTx derived the human cell type signature matrix from gene expression from 80% of the cells of each type. The remaining 20% of cells of each type were used to generate artificial mixture samples with different cellular compositions that were then used to test the signature. **(B)** Comparison between the proportions estimated by CIBERSORTx (observed) and those expected in the artificial mixture samples generated as in (A). **(C)** Comparison between the proportions estimated by CIBERSORTx (observed) and those expected in the artificial mixture samples generated as in (A) using the CIBERSORTx-derived mouse cell type signature. Pearson's correlation coefficients (r) between observed and expected proportions are shown for each cell type.

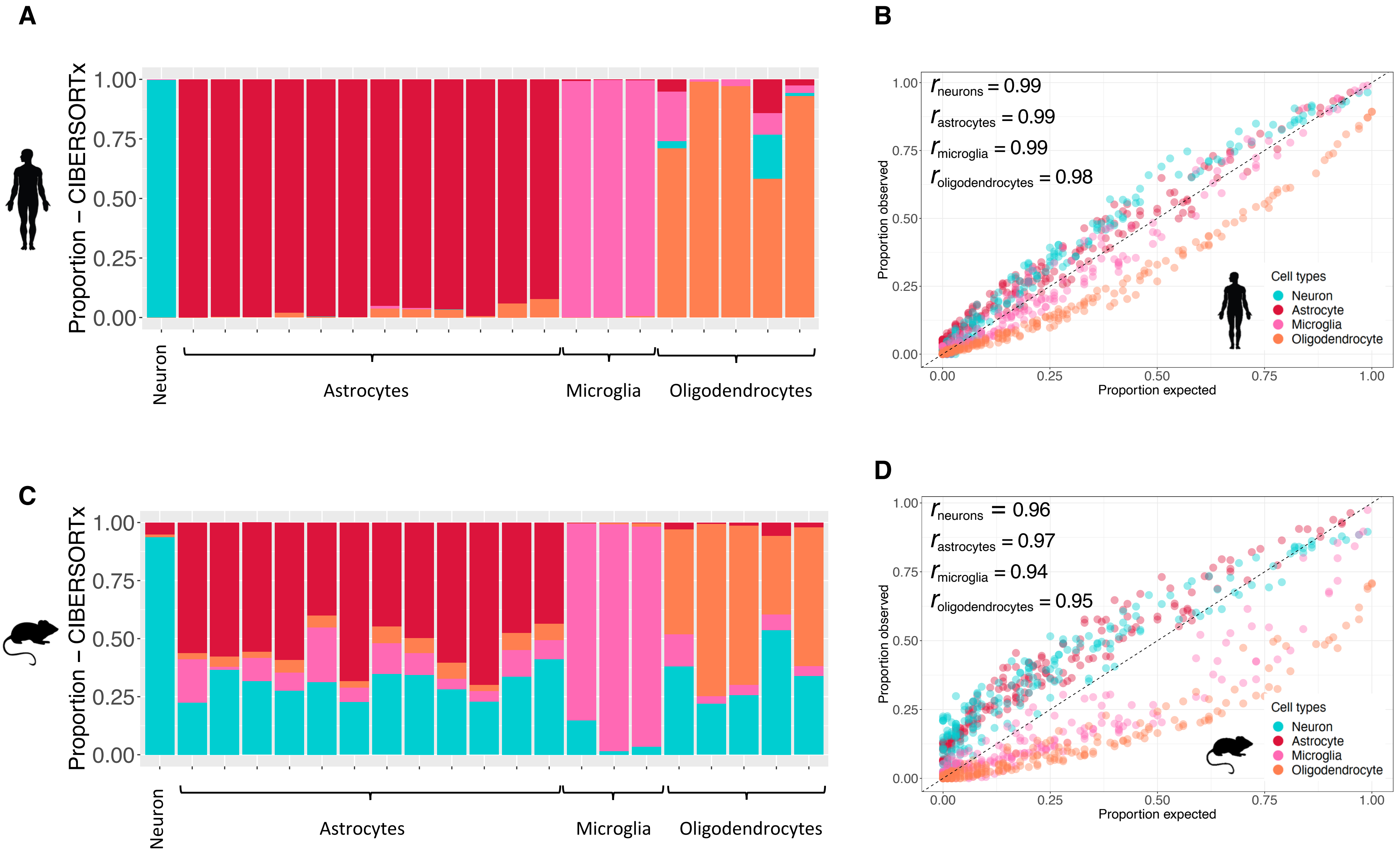

Figure S7 - Barplots of CIBERSORTx estimates of cellular composition of the Zhang single-cell samples based on the **(A)** human and **(C)** mouse cell type signatures. Comparisons between the proportions estimated by CIBERSORTx (observed) and those expected in the artificial mixture samples derived from the Zhang single-cell dataset, using the **(B)** human and **(D)** mouse cell type signatures. Pearson's correlation coefficients (r) between observed and expected proportions are shown for each cell type.

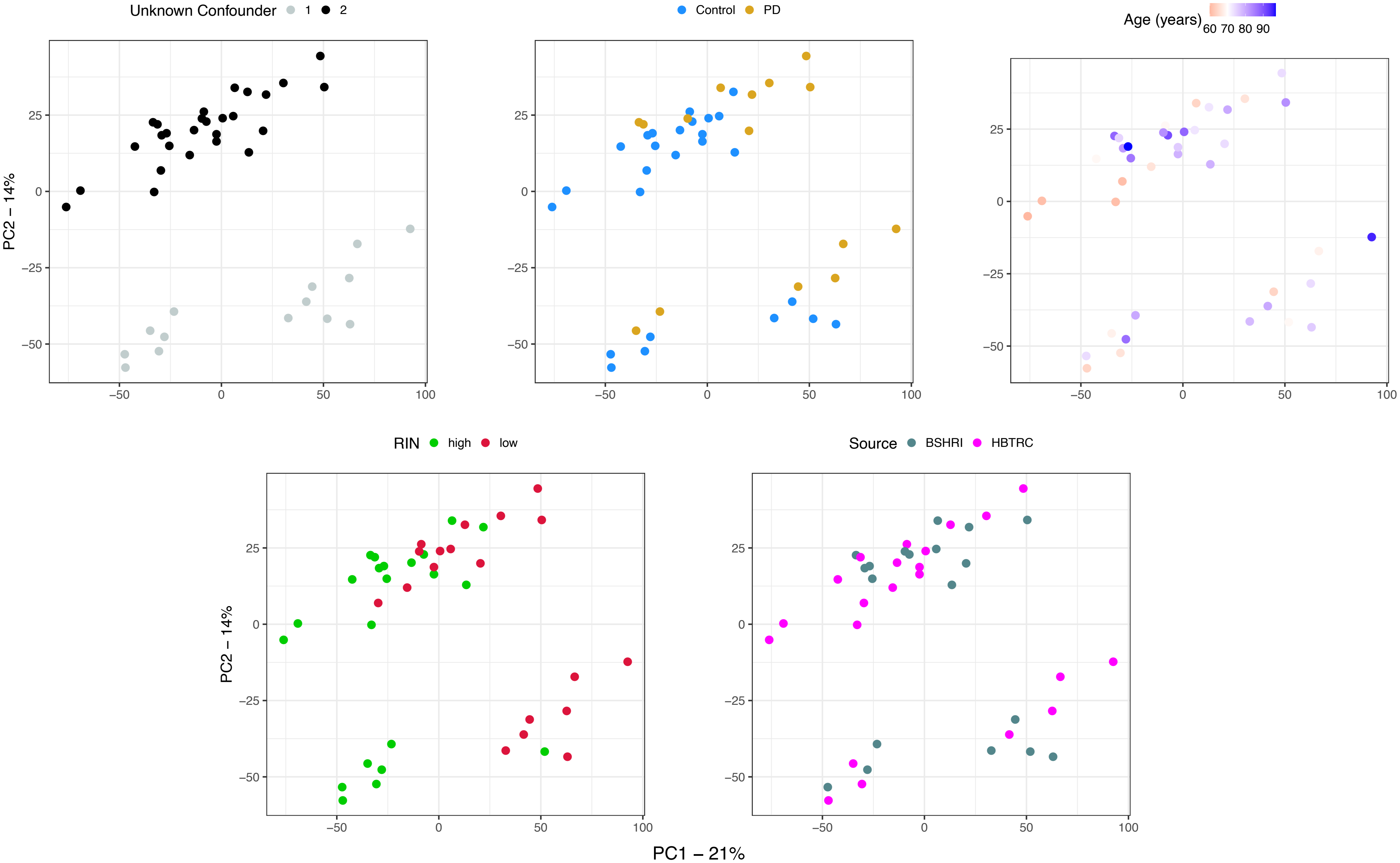

Figure S8 - Sample factorial maps of components 1 (PC1) and 2 (PC2) of Principal Component Analysis (PCA) of the gene expression in Dumitriu samples, with samples colored according to a different variable in each panel. Indicated in the respective axes labels are the percentages of data variance explained by the components. The confounder effect highlighted in the first panel could not be explained by any known variable, namely, condition (disease) status, age, RIN or source of the samples (second to fifth panels).
