# Supplementary material for: Unraveling Targetable Systemic and Cell-Type-Specific Molecular Phenotypes of Alzheimer’s and Parkinson’s Brains With Digital Cytometry
Source: Front Neurosci. 2020 Dec 9;14:607215. doi: 10.3389/fnins.2020.607215 (PMC7756021; doi:10.3389/fnins.2020.607215)
Supplement: Supplementary file 2 [file Data_Sheet_2.PDF]

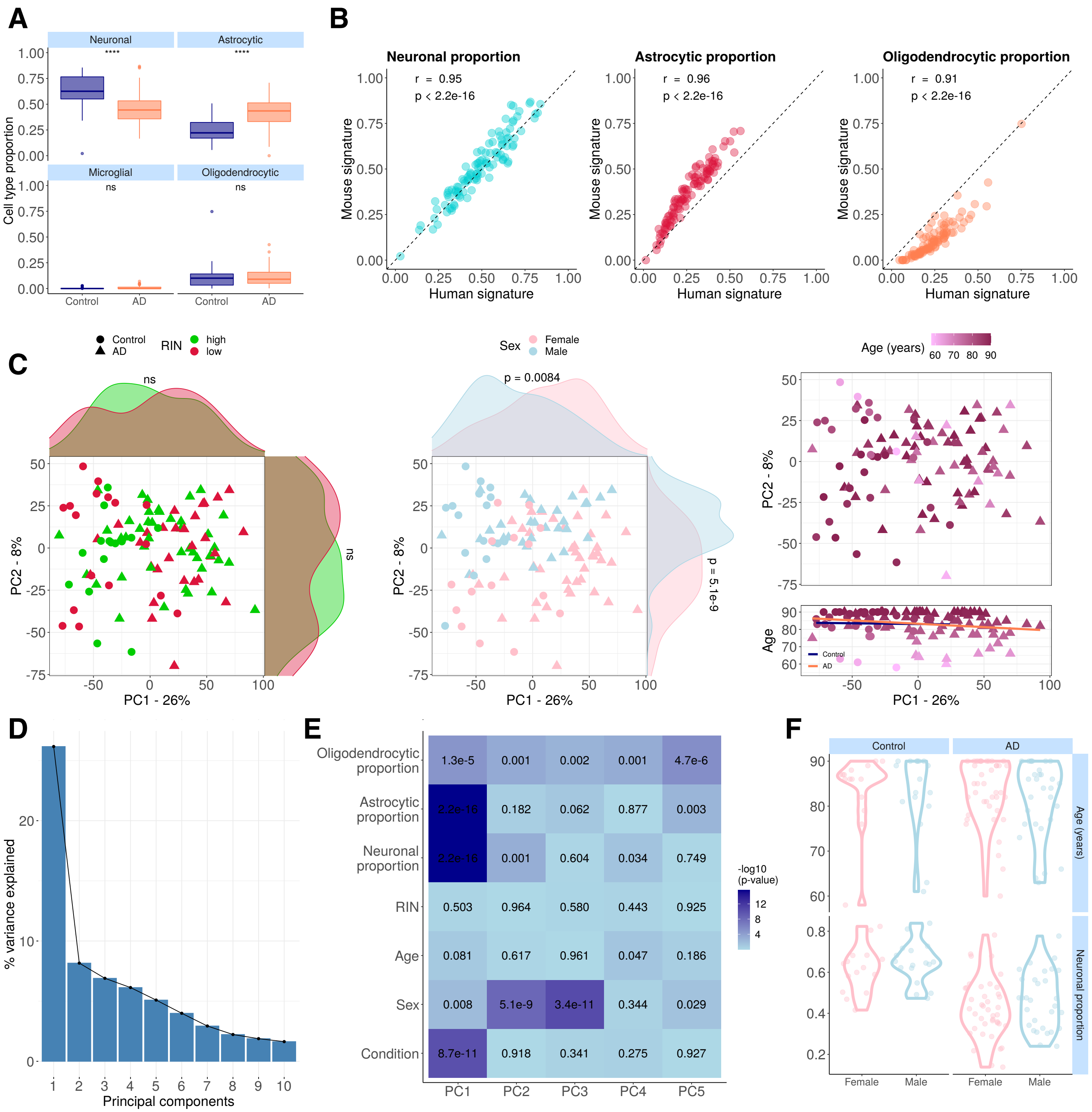

Figure S9 - (A) Estimates of the composition of MayoClinic samples in each main cell type based on the mouse cell type gene expression signature. Wilcoxon signed-rank tests were used to compare differences in proportions between Control and AD samples. ns: non-significant**,** ****: p <= 0.0001**. (B)** Comparison between estimated proportions of different cell types in MayoClinic samples obtained from the human and mouse cell type signatures. Their Pearson's correlation coefficient (r) and respective p-value (p) are shown. **(C)** Sample factorial map of components 1 (PC1) and 2 (PC2) of Principal Component Analysis (PCA) of the gene expression in MayoClinic samples, colored according to their RIN (left), Sex (middle) and Age (right). Indicated in the respective axes labels are the percentages of data variance explained by PC1 and PC2. Kolmogorov-Smirnov tests were used to compare the distributions of PC1 and PC2 loadings between RIN groups (left) and Sexes (middle), illustrated by the smoothed histograms along the respective axes of the PCA plots (ns: non-significant). In the lower right plot, the colored solid lines represent the linear regressions between age and PC1 loadings for AD and Control Samples. **(D)** Scree plot of the percentage of variance explained by each of the first 10 components of PCA. **(E)** Heatmap of significance, given by p-values, of the association between the loadings of each of the first 5 components of PCA and the seven annotated potentially explanatory sample variables. Spearman's correlation and Kolmogorov-Smirnov tests were used respectively for categorical (Condition, Sex and RIN) and continuous (proportions of astrocytes, neurons and oligodendrocytes, and Age) variables. **(F)** Violin plots of distributions of age and neuronal proportion of samples, discriminated between sexes and disease status.

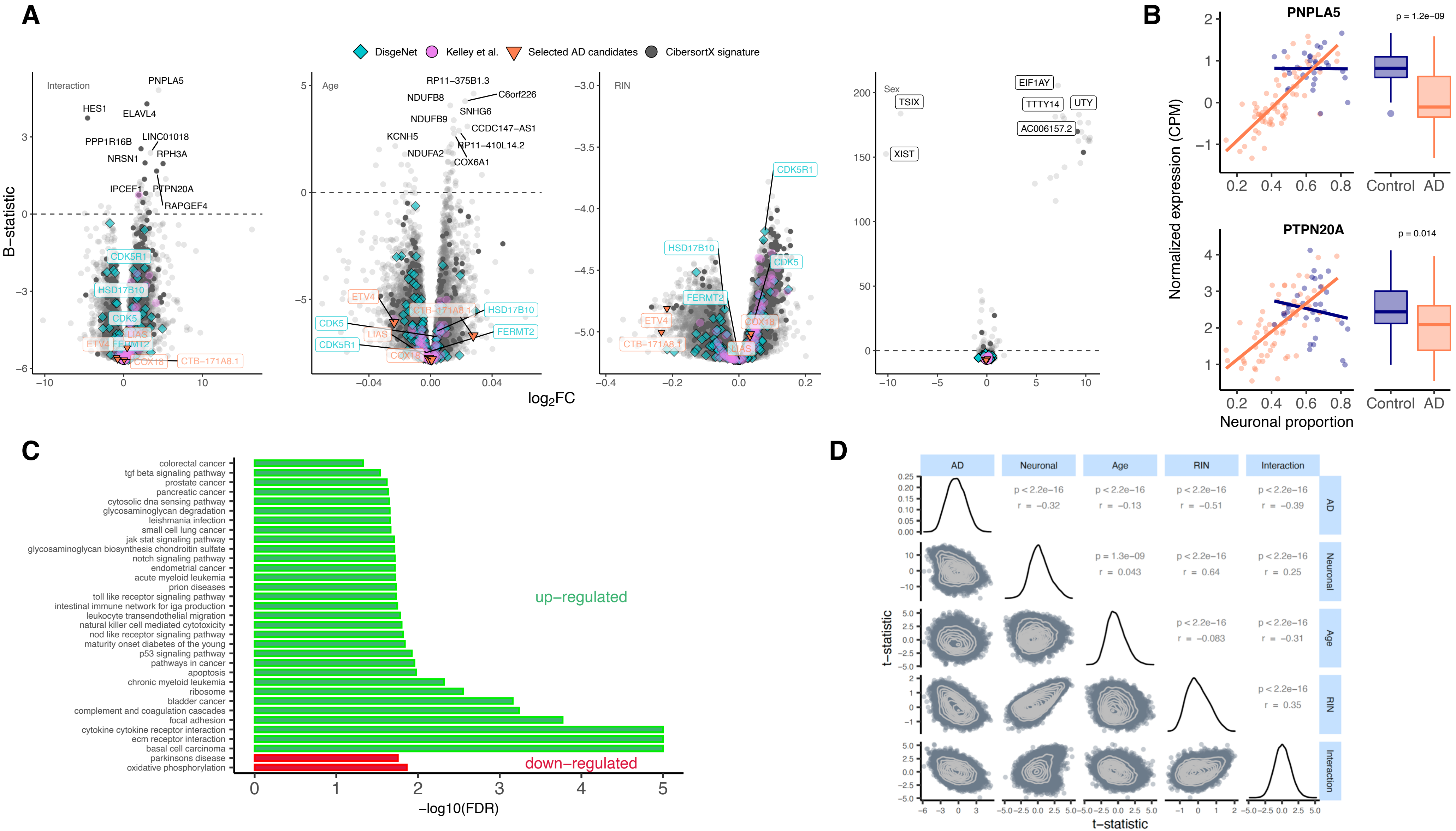

Figure S10 - **(A)** Volcano plots, relating log2 fold-changes ($\log_2$FC) and B-statistics, of differential gene expression associated with the Interaction, Age, RIN and Sex effects in MayoClinic samples. Highlighted with larger colored dots are disease-associated genes from DisgeNet (Piñero et al., 2019) (light blue), genes reported by (Kelley et al., 2018), as undergoing cell-type-specific changes in AD (pink), manually selected gene candidates for AD-specific alterations (orange), and genes included in the CIBERSORTx-derived expression signature for the major brain cell types (dark grey). Amongst all these, labelled are genes of particular interest, with individual expression profiles plotted in Figure 2B and 2C. The other labelled genes are the top 10 differentially expressed genes for Interaction and Age. Note that the most differentially expressed genes labelled in the Sex volcano plot lie on chromosomes X and Y, as expected. **(B)** Expression of genes selected as examples of high Interaction effect in Control and AD samples – scatterplots against neuronal proportion on the left, boxplots of distribution by condition on the right. T-tests, for which p-values are indicated, were used to compare gene expression mean differences between Control and AD samples. **(C)** Significance of enrichment of KEGG pathways in genes up-regulated (green) and down-regulated (red) in MayoClinic AD samples compared to Controls. **(D)** Below the diagonal: scatter plots (with light gray solid contour density lines) comparing the t-statistics of differential gene expression between pairs of modelled effects for the MayoClinic dataset. Diagonal: smoothed histograms of distributions of t-statistics of t-statistics of differential gene expression for the modelled effects. Above the diagonal: Pearson's correlation coefficients (r), and associated significance (p), of t-statistics of differential gene expression between pairs of modelled effects.

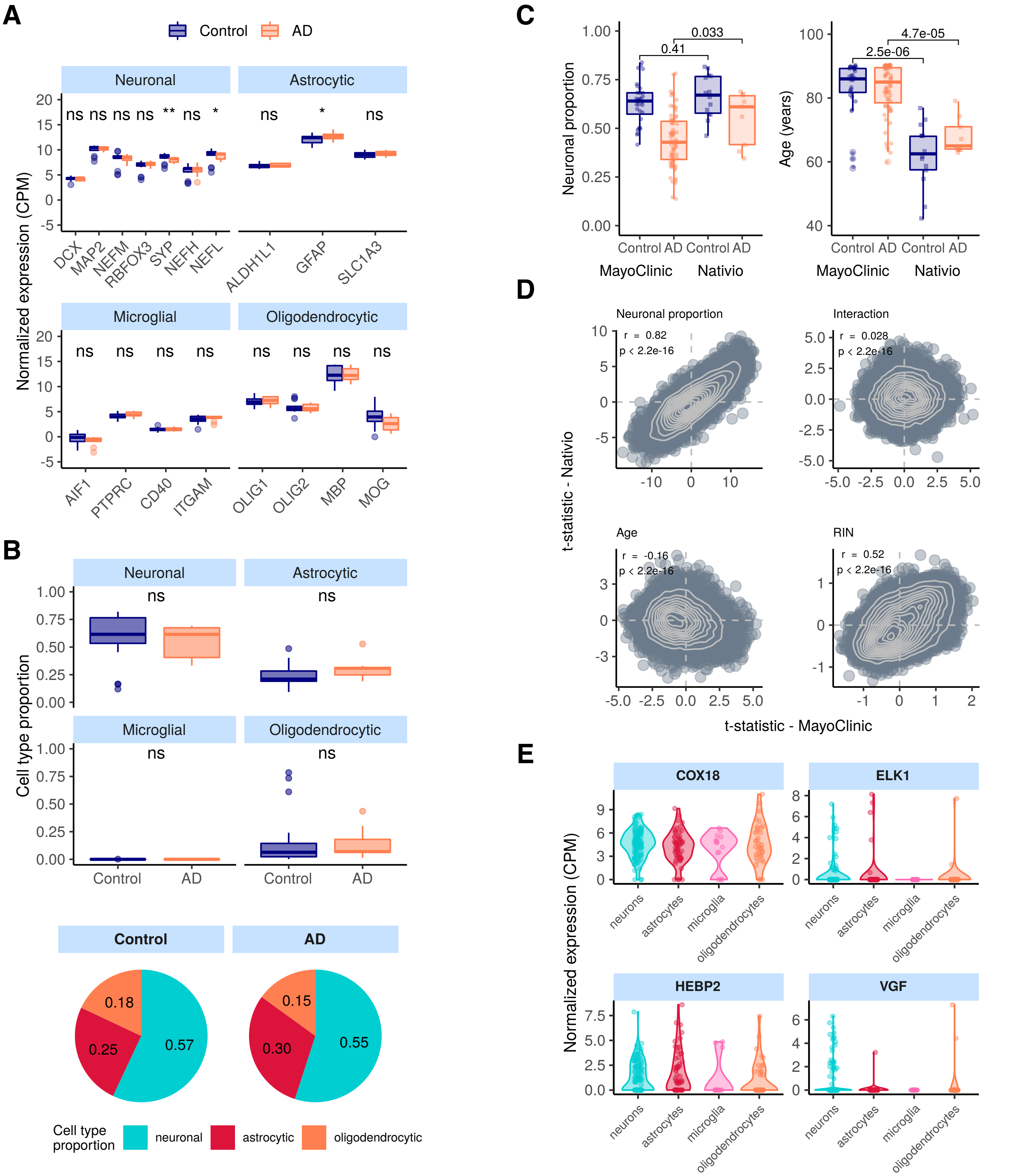

Figure S11 - (A) Neuronal, astrocytic, microglial and oligodendrocytic known markers' expression in the Nativio samples. T-tests were used to compare gene expression mean differences between Control and AD samples. **(B)** Estimates of the composition of Nativio samples in each main cell type based on the human cell type gene expression signature. Wilcoxon signed-rank tests were used to compare differences in proportions between Control and AD samples. **(C)** Boxplots of distributions of neuronal proportion and age of donors in Control and AD samples from the MayoClinic and Nativio datasets. Wilcoxon signed-rank tests were used to compare differences in neuronal proportion and age of samples in the same condition between datasets. **(D)** Scatter plots (with light gray solid contour density lines) comparing the t-statistics of differential gene expression of each modelled effect between the MayoClinic and the Nativio datasets. The respective Pearson's correlation coefficients (r) and associated p-values (p) are also indicated. **(E)** Violin plots of expression distributions of genes of interest in brain cells of the four main types from the Darmanis dataset.
Legend: ns: non-significant, *: p <= 0.05, ns: non-significant, **: p <= 0.01

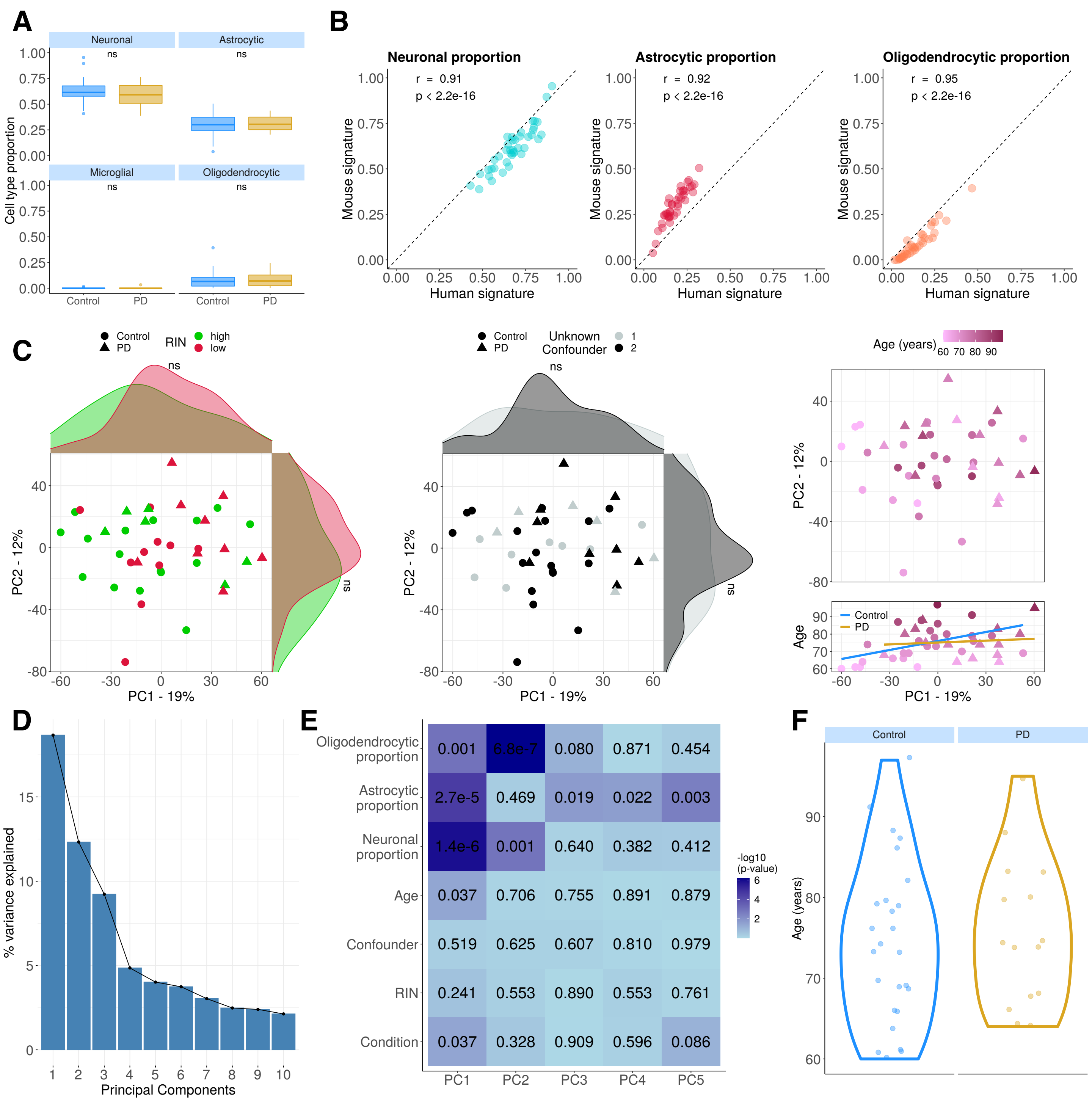

Figure S12 - (A) Estimates of the composition of Dumitriu samples in each main cell type based on the mouse cell type gene expression signature. Wilcoxon signed-rank tests were used to compare differences in proportions between Control and PD samples. ns: non-significant, ****: p <= 0.0001**. (B)** Comparison between estimated proportions of different cell types in Dumitriu samples obtained from the human and mouse cell type signatures. Their Pearson's correlation coefficient (r) and respective p-value (p) are shown. **(C)** Sample factorial map of components 1 (PC1) and 2 (PC2) of Principal Component Analysis (PCA) of the gene expression in Dumitriu samples, colored according to their RIN (left), Unknown confounder (middle) and Age (right). Indicated in the respective axes labels are the percentages of data variance explained by PC1 and PC2. Kolmogorov-Smirnov tests were used to compare the distributions of PC1 and PC2 loadings between RIN groups (left) and Unknown confounder (middle), illustrated by the smoothed histograms along the respective axes of the PCA plots (ns: non-significant). In the lower right plot, the colored solid lines represent the linear regressions between age and PC1 loadings for PD and Control Samples. **(D)** Scree plot of the percentage of variance explained by each of the first 10 components of PCA. **(E)** Heatmap of significance, given by p-values, of the association between the loadings of each of the first 5 components of PCA and the seven annotated potentially explanatory sample variables. Spearman's correlation and Kolmogorov-Smirnov tests were used respectively for categorical (Condition, Unknown confounder and RIN) and continuous (proportions of astrocytes, neurons and oligodendrocytes, and Age) variables. **(F)** Violin plots of distributions of age of samples, discriminated between disease status.

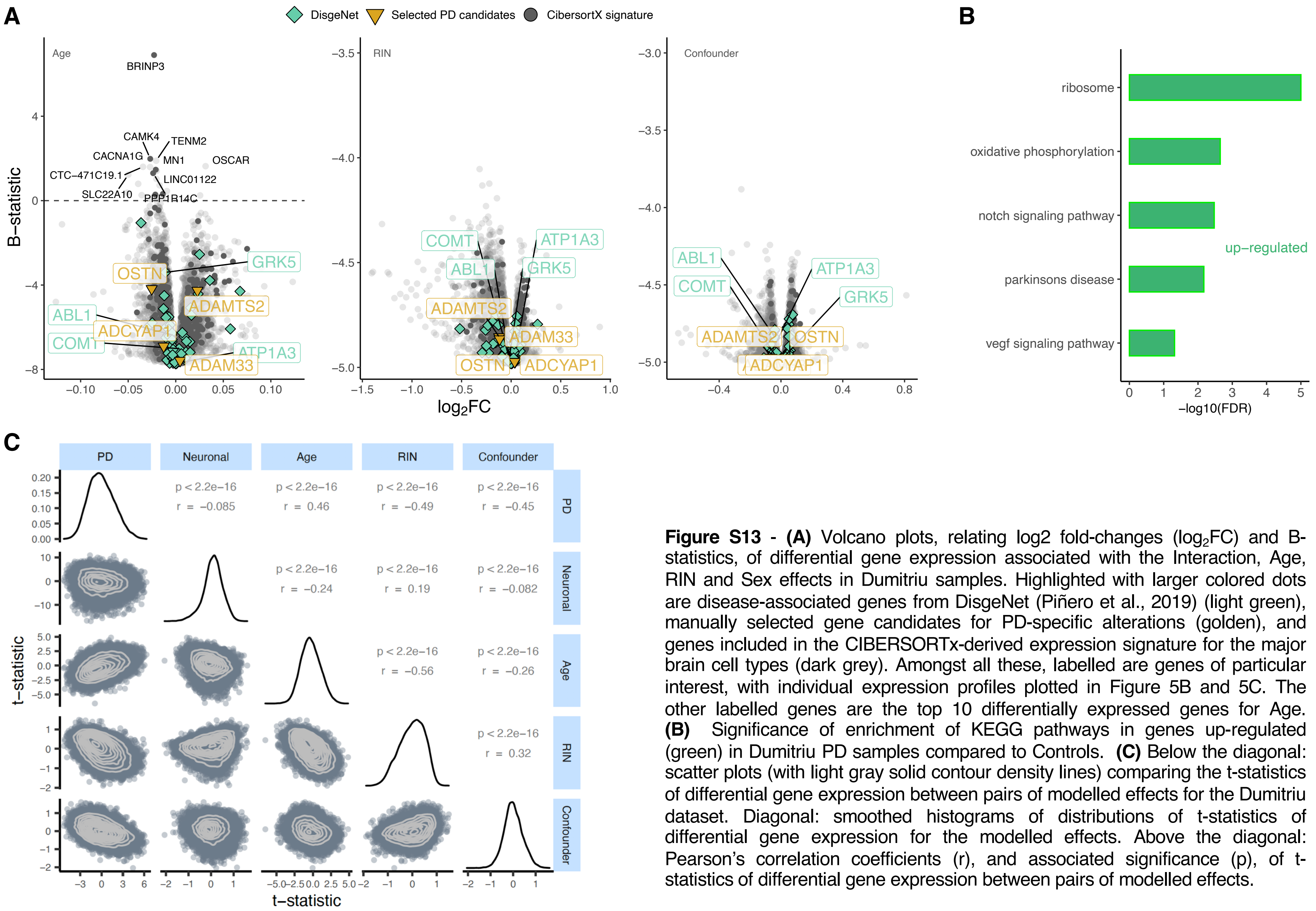

Figure S13 - **(A)** Volcano plots, relating log2 fold-changes (log_2FC) and B-statistics, of differential gene expression associated with the Interaction, Age, RIN and Sex effects in Dumitriu samples. Highlighted with larger colored dots are disease-associated genes from DisgeNet (Piñero et al., 2019) (light green), manually selected gene candidates for PD-specific alterations (golden), and genes included in the CIBERSORTx-derived expression signature for the major brain cell types (dark grey). Amongst all these, labelled are genes of particular interest, with individual expression profiles plotted in Figure 5B and 5C. The other labelled genes are the top 10 differentially expressed genes for Age. **(B)** Significance of enrichment of KEGG pathways in genes up-regulated (green) in Dumitriu PD samples compared to Controls. **(C)** Below the diagonal: scatter plots (with light gray solid contour density lines) comparing the t-statistics of differential gene expression between pairs of modelled effects for the Dumitriu dataset. Diagonal: smoothed histograms of distributions of t-statistics of differential gene expression for the modelled effects. Above the diagonal: Pearson's correlation coefficients (r), and associated significance (p), of t-statistics of differential gene expression between pairs of modelled effects.

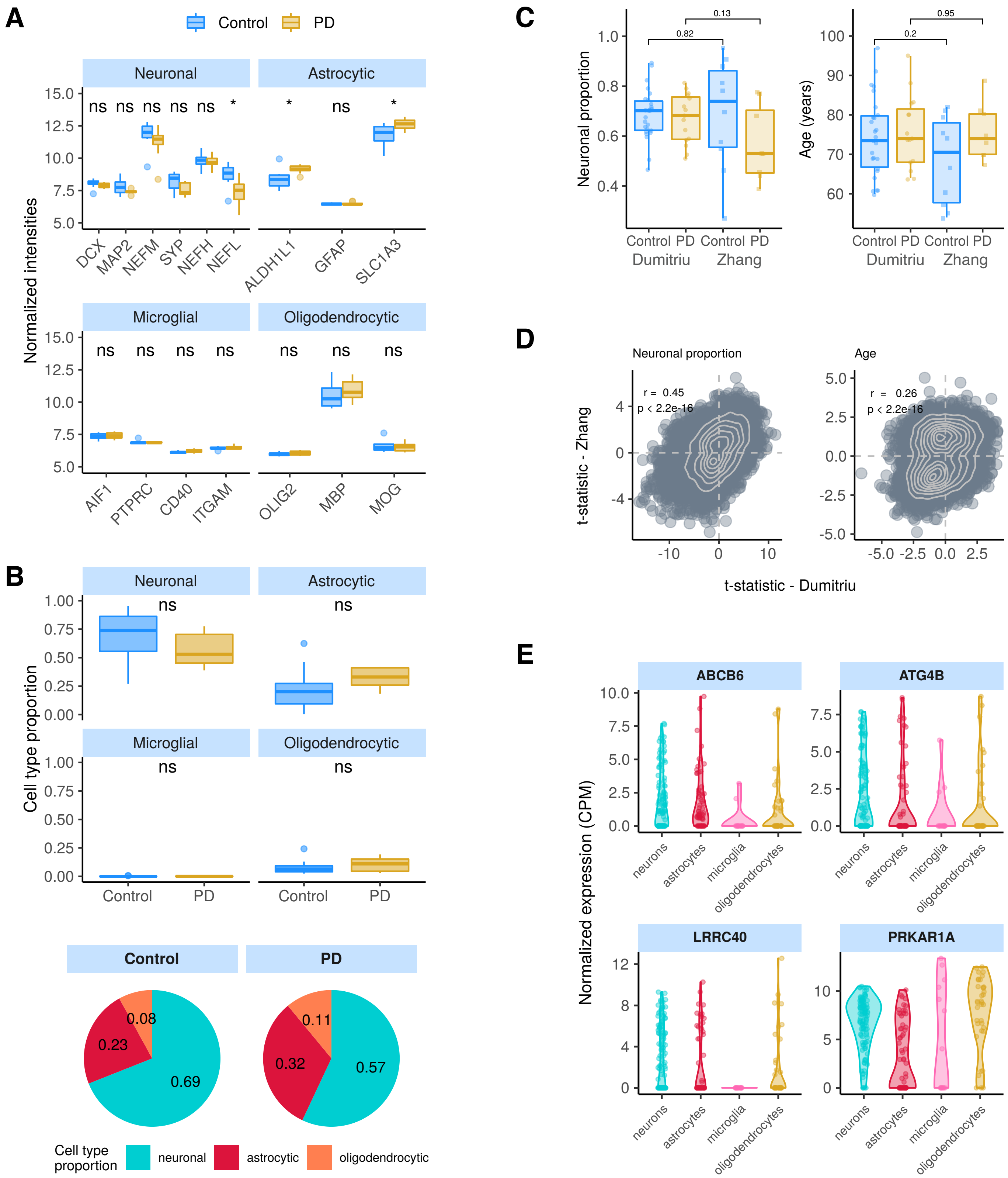

Figure S14 - (A) Neuronal, astrocytic, microglial and oligodendrocytic known markers' expression in the Zhang samples. T-tests were used to compare gene expression mean differences between Control and PD samples. **(B)** Estimates of the composition of Zhang samples in each main cell type based on the human cell type gene expression signature. Wilcoxon signed-rank tests were used to compare differences in proportions between Control and PD samples. **(C)** Boxplots of distributions of neuronal proportion and age of donors in Control and PD samples from the Dumitriu and Zhang datasets. Wilcoxon signed-rank tests were used to compare differences in neuronal proportion and age of samples in the same condition between datasets. **(D)** Scatter plots (with light gray solid contour density lines) comparing the t-statistics of differential gene expression of each modelled effect between the Dumitriu and the Zhang datasets. The respective Pearson's correlation coefficients (r) and associated p-values (p) are also indicated. **(E)** Violin plots of expression distributions of genes of interest in brain cells of the four main types from the Darmanis dataset.
Legend: ns: non-significant**,** *: p <= 0.05, ns: non-significant**,** **: p <= 0.01

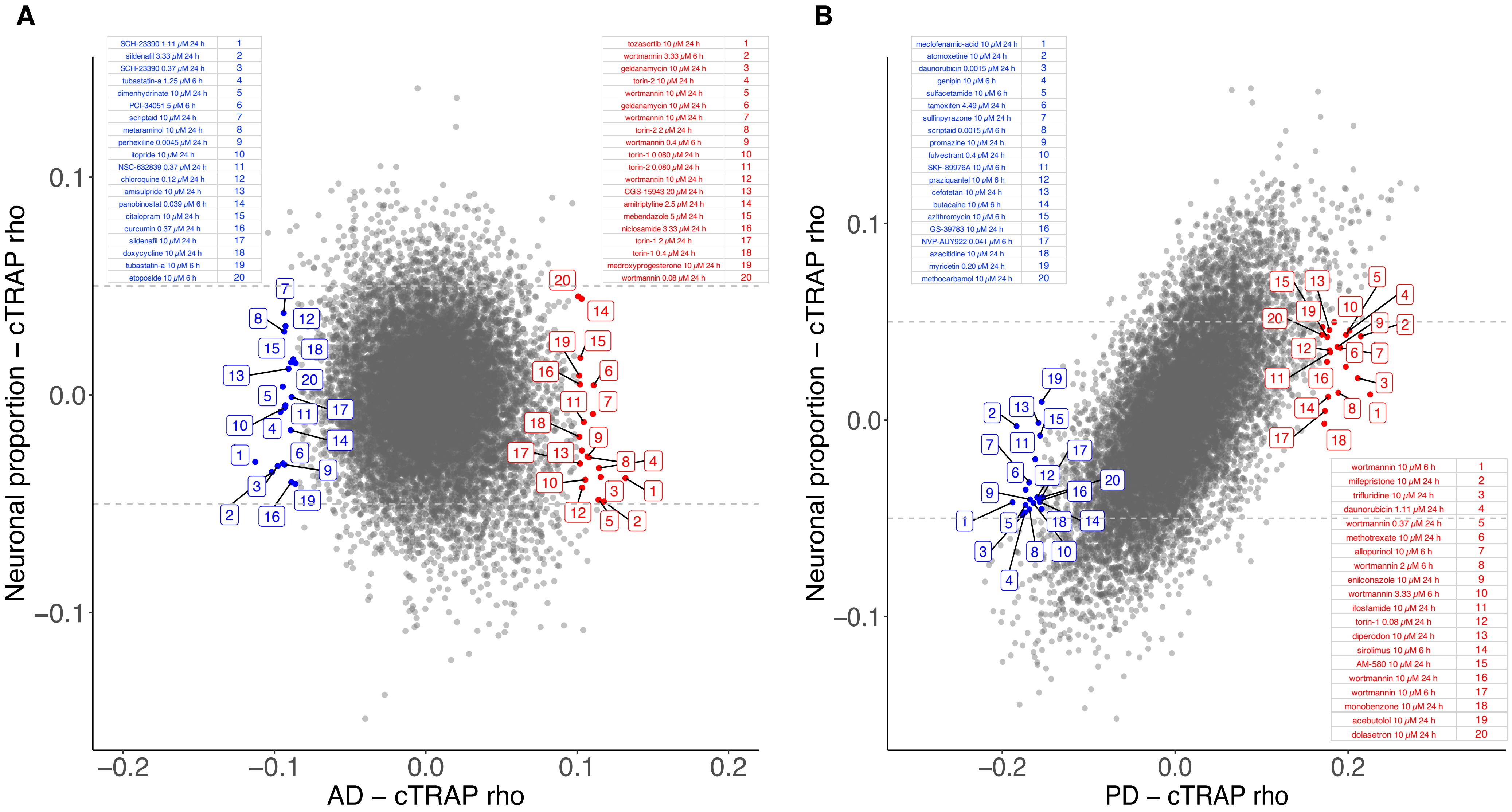

Figure S15 - (A) Scatter plot comparing, between the AD and the Neuronal proportion effects, the cTRAP-derived cross-gene Spearman's correlation coefficients (rho) of their differential expression combined scores with perturbation z-scores for cMap compounds. Labelled compounds are those selected as top candidates for reversal (blue) and induction (red) of AD-associated gene expression alterations (v. Materials and Methods) and with |rho| < 0.05 for Neuronal proportion (dashed light gray lines). **(B)** Scatter plot comparing, between the PD and the Neuronal proportion effects, the cTRAP-derived cross-gene Spearman's correlation coefficients (rho) of their differential expression combined scores with perturbation z-scores for cMap compounds. Labelled compounds are those selected as top candidates for reversal (blue) and induction (red) of PD-associated gene expression alterations (v. Materials and Methods) and with |rho| < 0.05 for Neuronal proportion (dashed light gray lines). Note: identical labels correspond to the same compound tested with the same concentration and at the same time point but in a different plate (v. Materials and Methods).
