# Supplementary material for: Unraveling Targetable Systemic and Cell-Type-Specific Molecular Phenotypes of Alzheimer’s and Parkinson’s Brains With Digital Cytometry
Source: Front Neurosci. 2020 Dec 9;14:607215. doi: 10.3389/fnins.2020.607215 (PMC7756021; doi:10.3389/fnins.2020.607215)
Supplement: Supplementary file 3 [file Data_Sheet_3.PDF]

Tables

Table S1 – CIBERSORTx estimates of the cellular composition of brain samples from all datasets.

- **Sample ID** is the sample's identifier.
- **Condition** is the sample's disease status (i.e., AD/PD or Control).
- **Neuronal proportion** is the proportion of neurons in the sample estimated by CIBERSORTx.
- **Astrocytic proportion** is the proportion of astrocytes in the sample estimated by CIBERSORTx.
- **Microglial proportion** is the proportion of microglia in the sample estimated by CIBERSORTx.
- **Oligodendrocytic proportion** is the proportion of oligodendrocytes in the sample estimated by CIBERSORTx.
- **Correlation** is the highest Pearson's correlation coefficient between gene expression profiles of artificial mixture samples, generated by CIBERSORTx using a comprehensive range of imputed cell fractions, the cell-type expression signature and the sample's gene expression profile. The estimated sample's cellular composition is indeed that of the most correlated artificial mixture sample.
- **RMSE** is the Root Mean Squared Error of the CIBERSORTx-modelled gene expression profile (i.e., that from the most correlated imputed artificial mixture) when compared to the actual (empirical) sample's gene expression.

Note: the p-values of the aforementioned correlation tests are not shown because they are all virtually zero.

Table S2 – Human brain cell types gene expression signature.

Relative expression, in arbitrary units (with 1 being the minimum), in the main human brain cell types of the genes selected by CIBERSORTx as able to discriminate between them.

Table S3 – Mouse brain cell types gene expression signature.

Relative expression, in arbitrary units (with 1 being the minimum), in the main murine brain cell types of the genes selected by CIBERSORTx as able to discriminate between them.

Table S4 – Artificial mixture samples.

Cell-type proportions of the 300 artificial mixture samples generated through chimeric libraries of 35 million reads, with numbers of randomly sampled read from the cell-type-specific pools also shown.

Table S5 – Modes of Action of cMap compounds.

Table S6 – Differential Gene Expression – MayoClinic dataset.
For each linearly modelled effect (i.e. each tab):

- **logFC** is the gene's log_2(fold-change) in expression associated with the effect.
- **AveExpr** is the gene's average expression (in log_2(CPM)) over all samples.
- **t** is the moderated t-statistic of differential gene expression.
- **P.Value** is the p-value associated with the t-statistic.
- **adj.P.Val** is the p-value associated with the t-statistic corrected for multiple testing by the Benjamini-Hochberg FDR procedure.
- **B** is B-statistic, i.e. the empirical Bayesian log-odds that the gene is differentially expressed.

Table S7 – Differential Gene Expression – Nativio dataset.
For each linearly modelled effect (i.e. each tab):

- **logFC** is the gene's log_2(fold-change) in expression associated with the effect.
- **AveExpr** is the gene's average expression (in log_2(CPM)) over all samples.
- **t** is the moderated t-statistic of differential gene expression.
- **P.Value** is the p-value associated with the t-statistic.
- **adj.P.Val** is the p-value associated with the t-statistic corrected for multiple testing by the Benjamini-Hochberg FDR procedure.
- **B** is B-statistic, i.e. the empirical Bayesian log-odds that the gene is differentially expressed.

Table S8 – Combined AD effect's scores from t-statistics of differential expression of common genes in the MayoClinic and Nativio datasets.

Table S9 – Combined Neuronal proportion effect's scores from t-statistics of differential expression of common genes in the MayoClinic and Nativio datasets.

Table S10 – Differential Gene Expression – Dumitriu dataset.

For each linearly modelled effect (i.e. each tab):

- **logFC** is the gene's log_2(fold-change) in expression associated with the effect.
- **AveExpr** is the gene's average expression (in log_2(CPM)) over all samples.
- **t** is the moderated t-statistic of differential gene expression.
- **P.Value** is the p-value associated with the t-statistic.
- **adj.P.Val** is the p-value associated with the t-statistic corrected for multiple testing by the Benjamini-Hochberg FDR procedure.
- **B** is B-statistic, i.e. the empirical Bayesian log-odds that the gene is differentially expressed.

Table S11 – Differential Gene Expression – Zhang dataset.

For each linearly modelled effect (i.e. each tab):

- **logFC** is the gene's log_2(fold-change) in expression associated with the effect..
- **AveExpr** is the gene's average expression (in log_2(CPM)) over all samples.
- **t** is the moderated t-statistic of differential gene expression.
- **P.Value** is the p-value associated with the t-statistic.
- **adj.P.Val** is the p-value associated with the t-statistic corrected for multiple testing by the Benjamini-Hochberg FDR procedure.
- **B** is B-statistic, i.e. the empirical Bayesian log-odds that the gene is differentially expressed.

Table S12 – Combined PD effect's scores from t-statistics of differential expression of common genes in the Dumitriu and Zhang datasets.

Table S13 – Combined Neuronal proportion effect's scores from t-statistics of differential expression of common genes in the Dumitriu and Zhang datasets.

Table S14 – Joint AD&PD Disease effect's scores from the combined AD and PD effects' scores.

Table S15 – Joint AD&PD Neuronal proportion effect's scores from the combined Neuronal proportion effects' scores from the AD and PD datasets.

Table S16 – cTRAP results: cMap compound perturbations and AD-associated gene expression changes.

- **cMAP perturbation** is the identifier of the chemical perturbation that incorporates information on the compound, cell line, time of exposure and dosage.
- **Compound** is the common name of the drug used.
- **Spearman's rho AD** is the correlation between the compound's perturbation z-scores and the AD differential expression combined scores.
- **Spearman's rho Neuronal porportion** is the correlation between the compound's perturbation z-scores and the Neuronal porportion differential expression combined scores.
- **Mode of Action** lists the compound's known modes of action.
- **Targets** are the known compound's gene targets.
- **Disease area** stands for the medical field in which the compound is already being administrated.
- **Phase** indicates the stage of clinical trials the drugs is in.

Table S17 – cTRAP results: cMap compound perturbations and PD-associated gene expression changes**.**

- **cMAP perturbation** is the identifier of the chemical perturbation that incorporates information on the compound, cell line, time of exposure and dosage.
- **Compound** is the common name of the drug used.
- **Spearman's rho PD** is the correlation between the compound's perturbation z-scores and the PD differential expression combined scores.
- **Spearman's rho Neuronal proportion** is the correlation between the compound's perturbation z-scores and the Neuronal proportion differential expression combined scores.
- **Mode of Action** lists the compound's known modes of action.
- **Targets** are the known compound's gene targets.
- **Disease area** stands for the medical field in which the compound is already being administrated.

- **Phase** indicates the stage of clinical trials the drugs is in.

Table S18 – cTRAP results: cMap compound perturbations and common AD- and PD-associated gene expression changes.

- **cMAP perturbation** is the identifier of the chemical perturbation that incorporates information on the compound, cell line, time of exposure and dosage.
- **Compound** is the common name of the drug used.
- **Spearman's rho AD&PD** is the correlation between the compound's perturbation z-scores and the AD-PD differential expression combined scores.
- **Mode of Action** lists the compound's known modes of action.
- **Targets** are the known compound's gene targets.
- **Disease area** stands for the medical field in which the compound is already being administrated.
- **Phase** indicates the stage of clinical trials the drugs is in.
